# Life in a rock pool: Radiation and population genetics of myxozoan parasites in hosts inhabiting restricted spaces

**DOI:** 10.1371/journal.pone.0194042

**Published:** 2018-03-21

**Authors:** Pavla Bartošová-Sojková, Alena Lövy, Cecile C. Reed, Martina Lisnerová, Tereza Tomková, Astrid S. Holzer, Ivan Fiala

**Affiliations:** 1 Institute of Parasitology, Biology Centre of the Czech Academy of Sciences, České Budějovice, Czech Republic; 2 Department of Biological Sciences, University of Cape Town, Rondebosch, South Africa; 3 Faculty of Science, University of South Bohemia in České Budějovice, České Budějovice, Czech Republic; National Cheng Kung University, TAIWAN

## Abstract

**Introduction:**

Intertidal rock pools where fish and invertebrates are in constant close contact due to limited space and water level fluctuations represent ideal conditions to promote life cycles in parasites using these two alternate hosts and to study speciation processes that could contribute to understanding the roles of parasitic species in such ecosystems.

**Material and methods:**

Gall bladder and liver samples from five clinid fish species (Blenniiformes: Clinidae) were morphologically and molecularly examined to determine the diversity, prevalence, distribution and host specificity of *Ceratomyxa* parasites (Cnidaria: Myxozoa) in intertidal habitats along the coast of South Africa. Phylogenetic relationships of clinid ceratomyxids based on the SSU rDNA, LSU rDNA and ITS regions were assessed additionally to the investigation of population genetic structure of *Ceratomyxa cottoidii* and subsequent comparison with the data known from type fish host *Clinus cottoides*.

**Results and discussion:**

Seven *Ceratomyxa* species including previously described *Ceratomyxa dehoopi* and *C*. *cottoidii* were recognized in clinids. They represent a diverse group of rapidly evolving, closely related species with a remarkably high prevalence in their hosts, little host specificity and frequent concurrent infections, most probably as a result of parasite radiation after multiple speciation events triggered by limited host dispersal within restricted spaces. *C*. *cottoidii* represents the most common clinid parasite with a population structure characterized by young expanding populations in the south west and south east coast and by older populations in equilibrium on the west coast of its distribution. Parasite and fish host population structures show overlapping patterns and are very likely affected by similar oceanographic barriers possibly due to reduced host dispersal enhancing parasite community differentiation. While fish host specificity had little impact on parasite population structure, the habitat preference of the alternate invertebrate host as well as tidal water exchange may be additional crucial variables affecting the dispersal and associated population structure of *C*. *cottoidii*.

## Introduction

**Fig 1:**
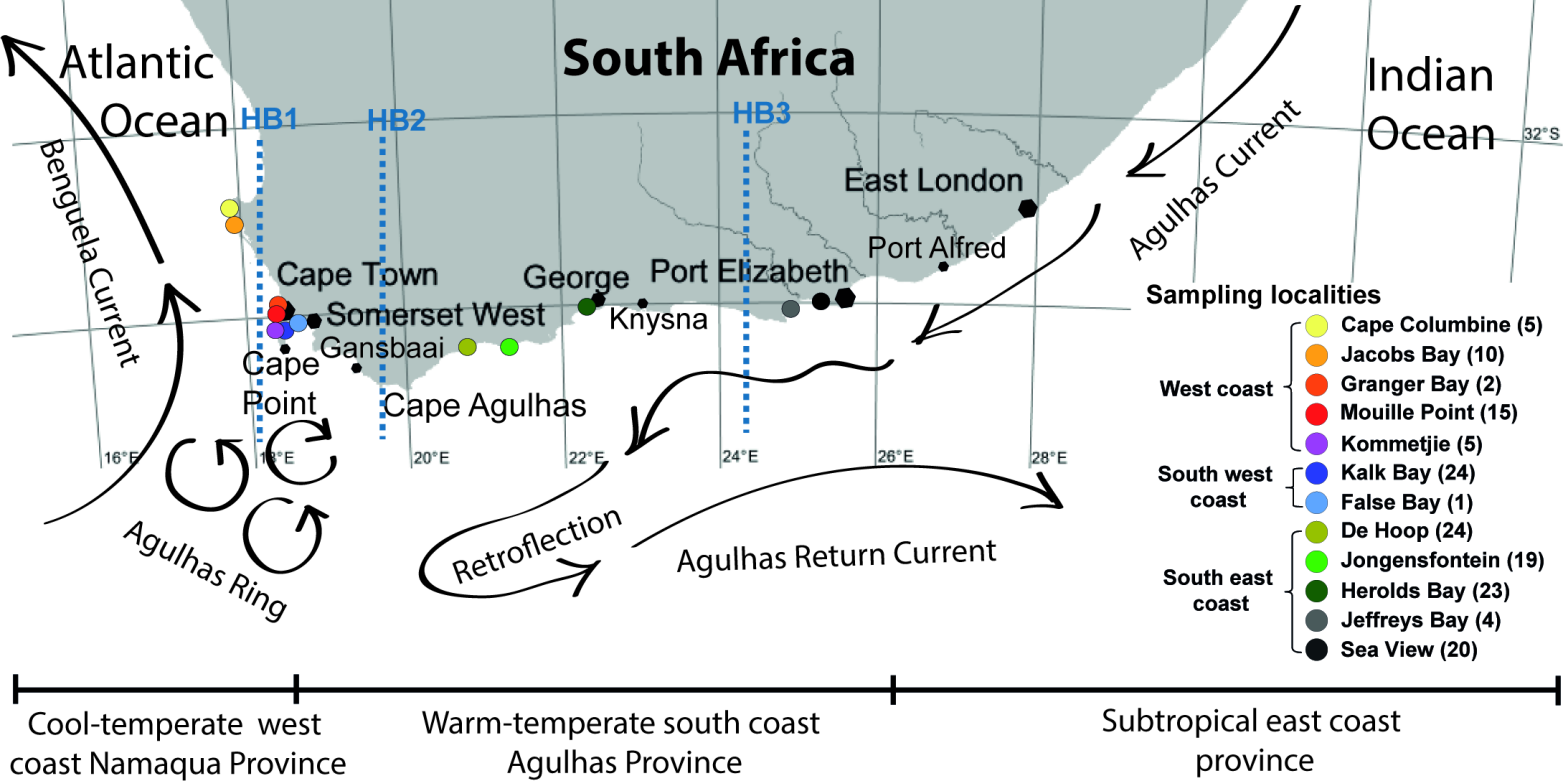
Map showing the sampling area of clinid fish in this study. The twelve sampling localities, grouped into three geographical regions, are labeled by round colored circles. The number of samples obtained at each locality is shown in parentheses. Moreover, the major oceanographic features around the South African coastline, the Benguela and Agulhas Currents, that influence population structuring of *Clinus cottoides* are shown along with the barriers to gene flow (blue vertical lines) as HB1: Jacobs Bay–Sea Point (at Cape Town), HB2: Gaansbai–Cape Agulhas, HB3: Knysna–Port Alfred [11, 12]. Agulhas ring eddies and the Agulhas counter-current are also highlighted.

Rock pools are unique and complex habitats in intertidal areas. They harbor large numbers of fishes and invertebrates that are well adapted to life in harsh conditions of strong fluctuations in water temperature, oxygen levels and salinity [1]. Fishes of the family Clinidae (Blenniiformes: Blennioidei), locally known as klipfish, are well adapted to such turbulent and unstable conditions [2, 3]. They are prominent inhabitants of these intertidal and shallow subtidal habitats and represent up to 98% of the total number of fish species in rock pool communities of South Africa [2, 3]. Distribution and genetic structure of South African clinids are strongly affected by oceanic circulation, paleoclimate changes, contemporary environmental variables, specific biological features and habitat preferences [4–6]. Significant biogeographic boundaries that affect South African marine fauna are created by two very distinct ocean currents that flank the west and south coasts. The Benguela Current, on the west coast, brings cold nutrient-rich water to the surface as it moves northwards along the west coast of the country. The Agulhas Current carries warm, nutrient-poor surface waters southwards along the east and south coasts of the country. A biogeographical barrier is created over the Agulhas Bank where these two currents meet (Fig 1) [4]. The latest glaciation events also affected the distribution of intertidal marine organisms in South Africa as a result of changes in sea levels and associated availability of rocky shore habitats [4–7].

The South African clinid genera *Clinus*, *Muraenoclinus*, *Blennophis*, and *Pavoclinus* as well as their sister-related Australian genera *Heteroclinus* and *Cristiceps* [8] are viviparous species giving birth to well-developed postflexion larvae [9]. This is in contrast to North and South American clinids which are oviparous [9]. Thus, the potential for free dispersal in South African live-bearing clinid fishes is highly reduced [10], creating more pronounced population genetic structures [6, 11, 12]. *Clinus cottoides* Valenciennes, 1836 (bluntnose klipfish) is a viviparous fish species with restricted dispersal capacity [10], which is endemic to South Africa and has a clear population structure with discontinuities along the oceanographic barriers that influence gene flow patterns. The first main genetic break lies on the southern South African coast (Gaansbai–Cape Agulhas; HB2 in Fig 1) while the second break is located between the east coast and other locations on the south (Knysna–Port Alfred; HB3 in Fig 1) [11]. Studies have also revealed additional pronounced genetic differentiation of *C*. *cottoides* populations between Jacobs Bay and Sea Point (HB1 in Fig 1) [12]. Similar isolation of populations on the east coast has been documented for the super klipfish *Clinus superciliosus* (L.) whereas a recent colonization event of the eastern range distribution has been suggested for the nosestripe klipfish *Muraenoclinus dorsalis* (Bleeker, 1860) [6, 12]. Both of these fish species represent species complexes showing evidence of cryptic speciation [6, 13].

Rock pools are important habitats in intertidal environments as they support a diverse assemblage of organisms [14, 15]. Moreover, these habitats represent ideal conditions for parasite life cycles as, due to the limited space, different host groups are in constant close contact [16]. The Myxozoa, a group of microscopic cnidarian parasites alternating mainly between the fish intermediate and annelid definitive hosts, are very common in marine habitats [17]. Representatives of five myxozoan genera *Henneguya*, *Ceratomyxa*, *Myxidium*, *Ortholinea* and *Sphaeromyxa* have so far been described from South African clinids [18–23]. *Ceratomyxa* is a species-rich genus characterized by crescent shaped spores with two polar capsules. Individual species mostly infect gall bladders and bile ducts of marine fishes and generally differ by spore measurements, host preference and SSU rRNA gene sequences [17, 24]. Ceratomyxids are highly host specific [25–30] and very few taxa infect more than one host species [25, 30]. Therefore, host as an informative diagnostic character has become particularly valuable for morphologically and genetically closely related species of *Ceratomyxa* [25–30]. Even though ceratomyxids in general cause little harm to their hosts, some species have been documented to induce severe histopathological damage [31–33] and obstruction and distension of bile ducts due to the presence of masses of spores and plasmodial stages [20]. The ceratomyxid life cycle has so far been elucidated only for *Ceratomyxa auerbachii* Kabata, 1962 which alternates between a clupeid fish and a marine polychaete [34]. Though globally distributed, the knowledge of *Ceratomyxa* species diversity in South African clinids is very limited. *Ceratomyxa obovalis* (Fantham, 1930) was found to parasitize blennioids *C*. *cottoides*, *C*. *superciliosus*, bull klipfish *Clinus taurus* Gilchrist & Thompson, 1908 and horned blenny *Parablennius cornutus* (L.) from False Bay and Kalk Bay [18, 19]. *Ceratomyxa cottoidii* Reed, Basson, Van As et Dyková, 2007 and *Ceratomyxa dehoopi* Reed, Basson, Van As et Dyková, 2007 were described from *C*. *cottoides* and *C*. *superciliosus*, respectively, from De Hoop [20], one of South Africa’s largest marine reserves. This area is located just east of Cape Agulhas, a dominant genetic break for marine intertidal organisms [6, 11, 35, 36]. A further undescribed *Ceratomyxa* species, morphologically similar to *C*. *cottoidii*, was reported from *M*. *dorsalis* from Kommetjie, Granger Bay and Jacobs Bay [37]. Interestingly, this species [37] and other ceratomyxids [26, 31, 38] display a high degree of morphological spore plasticity. This raises questions whether the observed variation is determined by deformations of their presumably thin-walled shell valves, a feature previously documented for some ceratomyxids [39], or alternatively is due to the concurrent infections of several *Ceratomyxa* species of different morphology, as mixed myxozoan infections [17], including those of *Ceratomyxa* spp. [40], are a common phenomenon.

Myxozoans are generally recognized as parasites with an accelerated rate of molecular evolution [41, 42] explained by an extraordinary level of radiation [43, 44]. Such rapid molecular evolution and associated long-branch attraction (LBA) can greatly obscure the results of phylogenetic studies of the Myxozoa [41]. Substitution rates significantly differ among myxozoan lineages. For example, the *Ceratomyxa* clade, placed within the marine myxosporean lineage [45, 46] includes several long-branching taxa sometimes clustering together, most probably due to LBA [46]. *Ceratomyxa* spp. from the same fish host family (and sometimes from the same geographic location) tend to cluster together, however, this is not always the rule [27, 29].

Though population structure of both, host and parasite, is most relevant to co-evolutionary processes [47–49] and myxozoan evolutionary history has been significantly linked to host-parasite co-evolution [50, 51], unfortunately, to date, investigations determining the population structure of myxozoan parasites in relation to that of their hosts are still missing. For this purpose, we selected the host-parasite model system of *Clinus cottoides*, rendering the detailed knowledge of its biology, distributional range, and population structure [10–12], and *Ceratomyxa cottoidii* for which information on its fish host species and distribution was collected during the previous [20] and this studies. Moreover, an ecological system with geographically limited host dispersion as seen in *C*. *cottoides* is ideal for study.

Our aims were to i) examine diversity, prevalence, distribution and host species spectra of ceratomyxids from South African clinids, ii) reconstruct the phylogenetic relationships among clinid ceratomyxids and to other members of the *Ceratomyxa* clade, and iii) reveal the patterns of population structuring of *C*. *cottoidii* and to investigate if they are linked to geographical or host-driven isolation of parasite populations. Based on previous knowledge, we hypothesized that ceratomyxids species in South African clinids are strictly host-specific, closely related and occur in mixed infections. Finally, we hypothesized that *C*. *cottoidii* as a specialist parasite follows the population structure of its fish host *C*. *cottoides*.

## Material and methods

### Sample collection, study area and time schedule

In total, 143 fish specimens of the family Clinidae (Perciformes) belonging to *Clinus acuminatus* (Bloch & Schneider), *Clinus brevicristatus* Gilchrist et Thompson, *C*. *cottoides*, *C*. *superciliosus* and *M*. *dorsalis* were collected from intertidal rock pools using small hand nets, at several localities in South Africa in March–April 2008, November 2009 and October–November 2012 (Fig 1, S1 Table). Fish were captured and treated under the terms and conditions of the MCM permit number RES2008/15, 2008/V8/LT and 2009/V1/LT of the Science Faculty Animal Ethics Committee (SFAEC) of University of Cape town (UCT) that approved this study. Captured fish were identified using Branch et al. [52].

### Processing of samples

Fish livers and gall bladders (in total 152 samples; S1 Table) were examined directly in the field or fixed in 70% ethanol and examined subsequently in the laboratory for the presence of myxosporean infections by light microscopy (LM) on Leica DM750 and Olympus BX51 microscopes. Plasmodia and myxospore morphology were documented with an Olympus DP70 digital camera using differential interference contrast. Samples were stored in 400 μL of TNES urea buffer (10 mM Tris-HCl with pH 8, 125 mM NaCl, 10 mM EDTA, 0.5% SDS and 4 M urea) or in 90% ethanol for subsequent DNA extraction.

### DNA extraction, PCR amplification, cloning and sequencing

Total DNA was extracted from all collected samples using a standard phenol-chloroform protocol, after an overnight digestion with proteinase K (50 μg mL^-1^; Serva, Germany), at 55°C. DNA was re-suspended in 50–100 μL^−1^ of DNAse-free water and left to dissolve overnight at 4°C.

Different regions of molecular markers within the nuclear ribosomal operon (SSU rDNA, LSU rDNA, ITS region) were amplified in this study with primer combinations as listed in Table 1. At the beginning we aimed at obtaining the sequence data of all three markers for *Ceratomyxa* parasites using the general *Ceratomyxa* (SSU rDNA) or general clinid *Ceratomyxa* (LSU rDNA, longer ITS region) primers from a random selection of several samples to compare the phylogenetic clustering patterns. Later on, we selected the longer ITS region as the most suitable marker for species detection based on the highest amount of data informativeness and best tree resolution and continued in PCRs of additional samples only for this region. Using these approaches, seven *Ceratomyxa* spp. were uncovered (*C*. *cottoidii*, *C*. *dehoopi*, further undescribed species *Ceratomyxa* sp. 1, *Ceratomyxa* sp. 2, *Ceratomyxa* sp. 3, *Ceratomyxa* sp. 4 and *Ceratomyxa* sp. 5). However, amplification of all three loci from samples chosen for amplification of all markers was not successful in all cases. This situation was further complicated by identification of concurrent infections of multiple *Ceratomyxa* spp. from clones placed in different clinid *Ceratomyxa* subclades (representing species) originating from single PCR products. To overcome these obstacles, which would bias the exact assessment of diversity and prevalence of *Ceratomyxa* spp., we designed seven sets of species- (lineage) specific primers targeting the shorter ITS region of each *Ceratomyxa* species (Table 1). This region was nested within the previously amplified longer ITS region and provides enough interspecific sequence variation and sufficient number of sequences for primer design. We subsequently PCR-screened all 152 samples.

**Table 1:** Primers used to amplify target regions of the nuclear ribosomal operon of clinid *Ceratomyxa* spp.

Primer name	Primer sequence	Target group/species	PCR type/ Amplicon length (bp)	Reference
**SSU rDNA**				
ERIB1	ACCTGGTTGATCCTGCCAG	Universal eukaryotic	Primary PCR ≈ 1900–2050	[53]
ERIB10	CTTCCGCAGGTTCACCTACGG	Universal eukaryotic		[53]
18S-cerF	CTWGTTGGTADGGTAGTG	All *Ceratomyxa*	Nested PCR ≈ 1300–1450	[46]
18S-cerR	GTACAAGAGGCAGAGACGTAT	All *Ceratomyxa*		[46]
**LSU rDNA**				
NLF-CerCot-out	ACCGTGATTGTCCCAGTAACTGCG	Clinid *Ceratomyxa*	Primary PCR ≈ 1050–1150	This study
NLR-CerCot-out	AAAGTGGCCCACTTGGAGCGC	Clinid *Ceratomyxa*		This study
NLF-CerCot-in	ACAAGAGCCCGTAGTCGAATCGC	Clinid *Ceratomyxa*	Nested PCR ≈ 950–1050	This study
NLR-CerCot-in	TTAGGAGCCTGCTCGCCGGTTGGG	Clinid *Ceratomyxa*		This study
**Longer ITS region (last 50 bp of SSU rDNA, full ITS1, full 5.8S rDNA, full ITS2, first 85 bp of LSU rDNA)**				
CerCot_ITS_for	CACGGCGACGGTGTGAAAACG	Clinid *Ceratomyxa*	Primary PCR ≈ 700–1050	This study
C_cot-ITS-R-out	GCGATTCGACTACGGGCTCTTGT	Clinid *Ceratomyxa*		This study
C_Cot-ITS-F-in	GAAGTAAAAGTCGTAACAAGG	Clinid *Ceratomyxa*	Nested PCR ≈ 500–850	This study
C_Cot-ITS-R-in	CGCAGTTACTGGGACAATCACGGT	Clinid *Ceratomyxa*		This study
**Shorter ITS region (cca 60–300 bp of ITS1 + 5.8S rDNA + cca 60–100 bp of ITS2)**				
C_Cot-ITS-F-in	GAAGTAAAAGTCGTAACAAGG	Clinid *Ceratomyxa*	Primary PCR ≈ 500–850	This study
C_Cot-ITS-R-in	CGCAGTTACTGGGACAATCACGGT	Clinid *Ceratomyxa*		This study
CCotITS162-for	TACGRKWRVARGTACTKTGAGGATG	*C*. *cottoidii*	Nested PCR ≈ 300	This study
CCotITS730-rev	TTTTMCGGCAGAGTAACC	*C*. *cottoidii*		This study
CDehITS154-for	AGAGTRCGTWTGGTGCGTTGAAG	*C*. *dehoopi*	Nested PCR ≈ 300	This study
CDehITS789-rev	TCGAGTCGGGTCAGTTGTTGTTC	*C*. *dehoopi*		This study
Csp1ITS223-for	AGTGTAAATATATGSCTACMTG	*Ceratomyxa* sp. 1	Nested PCR ≈ 300	This study
Csp1ITS801-rev	TTCATCAAGGGCAAATGTCGTC	*Ceratomyxa* sp. 1		This study
Csp2ITS315-for	AAGAGTAAAGATGAATGCCTAC	*Ceratomyxa* sp. 2	Nested PCR ≈ 350	This study
Csp2ITS824-rev	TTGTAATTTACAGGGGTCTAGG	*Ceratomyxa* sp. 2		This study
Csp3ITS198-for	TGAACGAAAGATGAAWTWAAGC	*Ceratomyxa* sp. 3	Nested PCR ≈ 350–550	This study
Csp3ITS785-rev	TGTTACTTGGTTGCCAGTCAG	*Ceratomyxa* sp. 3		This study
Csp4ITS199-for	AKGTRGACTGTGCTGCTTGCAG	*Ceratomyxa* sp. 4	Nested PCR ≈ 350	This study
Csp4ITS798-rev	TTCTAGTTGTCGGGYTTGTACCG	*Ceratomyxa* sp. 4		This study
Csp5ITS151-for	AGGTGAAATGGTGCAGTCTATC	*Ceratomyxa* sp. 5	Nested PCR ≈ 350	This study
Csp5ITS782-rev	TTCACCGTAACCATCATTCAC	*Ceratomyxa* sp. 5		This study

PCRs of the first three regions were carried out in a total volume of 25 μl consisting of 1x Taq Buffer, 250 μM of each dNTP, 10 pmol of each primer, 1 U of Taq-Purple polymerase (Top-Bio, Czech Republic), 1 μl of DNA (50 to 150 ng) and sterile distilled H_2_O. For the PCR screening based on the shorter ITS region, an AmpOne HS-Taq premix (GeneAll Biotechnology, South Korea) was used to prepare PCR reactions containing 10 μl of HS-Taq premix, 0.5 μl of each primer (25 pmol), 8 μl of sterile distilled H_2_O and 1 μl of DNA (50 to 150 ng). The following cycling parameters were used for these primary/nested PCRs i) SSU rDNA: 95°C 3 min, 30x (95°C 1 min, 52°C/55°C 1 min, 72°C 1 min 40s/1 min 15s), 72°C 7 min, ii) LSU rDNA: 95°C 3 min, 30x (95°C 30 s, 50°C/55°C 30s, 72°C 1 min), 72°C 7 min, iii) longer ITS region: 95°C 3 min, 30x (95°C 30 s, 50°C/56°C 30s, 72°C 1 min/40 s), 72°C 7 min, and iv) shorter ITS region: 95°C 3 min, 30x (95°C 30 s, 56°C/55°C 30s, 72°C 40 s/30s), 72°C 7 min.

PCR products were purified using a Gel/PCR DNA Fragments Extraction Kit (Geneaid Biotech Ltd, USA) and sequenced directly. In most cases, the amplified fragments were cloned into the pDrive Vector with a PCR Cloning Kit (Qiagen, Germany) and transformed into TOP10 chemically competent *Escherichia coli* cells (Life Technologies, Czech Republic). Plasmid DNA was extracted using a High Pure Plasmid Isolation Kit (Roche Applied Science, Germany) and up to 1–8 (mostly three) colonies (clones) were sequenced (SeqMe, Czech Republic).

A table with detailed information on sample localities and fish species, DNA extracted, numbers of PCR products and clones sequenced for each locus of a particular *Ceratomyxa* species is supplemented (S1 Table).

### Sequence alignments

Five datasets were created for the phylogenetic analyses and the haplotype network: i) *Ceratomyxa*-SSU rDNA dataset (1176 bp) composed of 70 sequences representing seven newly sequenced *Ceratomyxa* spp. from clinids (2 sequences for each species, in total 14 sequences), other selected members of the *Ceratomyxa* clade retrieved from the GenBank (54 sequences) and two outgroup sequences (basal ceratomyxids *Ceratomyxa leatherjacketi* and *Myxodavisia bulani*) ii) clinid-*Ceratomyxa*-SSU rDNA dataset (1465 bp) including 106 SSU rDNA sequences of seven newly sequenced *Ceratomyxa* spp. from clinids (104 sequences) and two outgroup sequences (*Ceratomyxa longipes* and *Ceratomyxa barnesi*), iii) clinid-*Ceratomyxa*-LSU rDNA dataset (1068 bp) including 122 LSU rDNA sequences of five newly sequenced *Ceratomyxa* spp. from clinids (118 sequences) and four outgroup sequences (*Ceratomyxa appendiculata*, *Ceratomyxa cardinalis*, *Ceratomyxa vikrami*, *Ceratomyxa verudaensis*), iv) clinid-*Ceratomyxa*-ITS dataset (908 bp) including 173 ITS sequences of seven newly sequenced *Ceratomyxa* spp. from clinids (170 sequences) and three outgroup sequences (*Ceratonova shasta*, *Cystodiscus australis*, *Parvicapsula* sp.), and v) *Ceratomyxa cottoidii*-ITS haplotype dataset (264 bp) including 76 new ITS sequences of *C*. *cottoidii*. This dataset, prepared for the haplotype networks and population genetics analyses, contained sequences from both the longer and the shorter ITS region that were trimmed to the length of the shortest ones (see Table 1). Nucleotide sequences in each dataset were aligned in MAFFT v7.017 algorithm [54] implemented in Geneious v8.0.5 [55] using the E-INS-i multiple alignment method, with a gap opening penalty (–op) of 1.53 and gap extension penalty (–ep) of 0.123. The alignments were manually edited and highly variable sections were removed manually in Geneious.

### Phylogenetic analyses

Maximum parsimony (MP) analysis was performed in PAUP* v4.b10 [56], using a heuristic search with random taxa addition, the ACCTRAN option, TBR swapping algorithm, all characters treated as unordered and gaps treated as missing data. Maximum likelihood (ML) analysis was performed in RAxML v7·0·3 [57] using the GTR + Γ model of nucleotide substitution. Bootstraps were based on 1000 replicates for both analyses. Bayesian inference (BI) analysis was performed in MrBayes v3.0 [58], using the GTR + Γ + I model of evolution. Analyses were initiated with random starting trees, four simultaneous MCMC chains sampled at intervals of 100 trees and posterior probabilities estimated from 1 million generations for the *Ceratomyxa*-SSU rDNA dataset (burn-in 100,000), 3 million generations for the clinid-*Ceratomyxa*-SSU rDNA dataset (burn-in 600,000), 3.5 million generations for the clinid-*Ceratomyxa*-LSU rDNA dataset (burn-in 350,000) and the clinid-*Ceratomyxa*-ITS dataset (burn-in 710,000). Suitable burn-in levels were chosen in Tracer v1.4.1 [59].

### Distances

The intraspecific and interspecific divergences were determined from proportional distances (in %) which were calculated in Geneious from the datasets previously used for the phylogenetic analyses. These datasets were additionally adjusted by excluding the very short sequences and by trimming the 5’ and 3’ ends of the remaining sequences to achieve their same length. The intraspecific cut-offs for the studied markers in the group of long branching ceratomyxids from clinid hosts were as follows: SSU rDNA–7.5%, LSU rDNA–10%, ITS region–20%.

### Haplotype networks, AMOVA and population genetic statistics

The ITS region (*Ceratomyxa cottoidii*-ITS haplotype dataset, 76 sequences) was selected for haplotype networks, analysis of molecular variance (AMOVA) and population genetic statistics, as it provides enough sequence variability to distinguish the population structure patterns in myxozoans [60].

To examine the evolutionary relationships among haplotypes in populations of *C*. *cottoidii* from different localities and clinid fish species statistical parsimony networks (TCS) based on pairwise differences were constructed using PopART v1.7 [61]. Sampling localities were set as traits for the haplotype network which grouped the sequences (number of sequences = n) with regard to geography (Cape Columbine n = 6, Jacobs Bay n = 2, Granger Bay n = 1, Mouille Point n = 4, Kommetjie n = 1, Kalk Bay n = 24, False Bay n = 1, De Hoop n = 14, Jongensfontein n = 2, Herolds Bay n = 19, Jeffreys Bay n = 2). Fish host species were set as traits for the haplotype network which grouped the sequences with regard to host specificity (*C*. *cottoides* n = 61, *C*. *superciliosus* n = 9, *C*. *acuminatus* n = 1, *C*. *brevicristatus* n = 1, *M*. *dorsalis* n = 4).

Population structure was estimated by AMOVA in PopART. Individuals represented by ITS region sequences were grouped by sampling locality to populations and then nested within groups delimited by putative oceanographic barriers and host-driven isolation. We performed AMOVAs at three different levels to quantify how much variation is partitioned: (i) among individuals within populations, (ii) among populations within groups, and (iii) among groups of populations. *A priori* groupings were as follows: i) “Geographic groups” formed according to the geographic separation of the South African coastline [11]: “West coast group” (n = 14): localities Cape Columbine, Jacobs Bay, Granger Bay, Mouille Point, Kommetjie; “South west coast group” (n = 25): Kalk Bay, False Bay; “South east coast group” (n = 37): De Hoop, Jongensfontein, Herolds Bay, Jeffreys Bay, ii) “Barrier groups” formed in accordance with the main barriers to gene flow recognized for the populations of the type fish host *Clinus cottoides* (HB1, HB2 and HB3 in Fig 1) [11, 12] with an exception that the most eastern locality, Jeffreys Bay, was not analyzed as a separate group due to low sample size (n = 2), so it was merged into a single group with the south coast localities De Hoop, Jongensfontein and Herolds Bay: “B1 group” (n = 8): Cape Columbine, Jacobs Bay; “B2 group” (n = 31): Kalk Bay, False Bay, Granger Bay, Mouille Point, Kommetjie; “B3 group” (n = 37): De Hoop, Jongensfontein, Herolds Bay, Jeffreys Bay, and iii) “Host groups” formed according to fish host species: “Clinus cottoides” (n = 61): *C*. *cottoides* from Kalk Bay, De Hoop, Jongensfontein, Herolds Bay, Jeffreys Bay; “Clinus superciliosus” (n = 9): *C*. *superciliosus* from Cape Columbine, Mouille Point, Kommetjie; and “Muraenoclinus dorsalis” (n = 4): *M*. *dorsalis* from Jacobs Bay, Granger Bay and Mouille Point. The variation was not quantified for *C*. *acuminatus* and *C*. *brevicristatus* due to small sample size (1 sequence for each host species).

To characterize the diversity of populations and their demographic history we performed several population genetic statistics for *C*. *cottoidii* groups that showed significant variation among the groups tested in the previous AMOVA (i.e. above defined geographic and barrier groups). DNA polymorphism statistics (number of haplotypes, number of segregating sites, nucleotide genetic diversity, haplotype diversity) and statistical tests aiming at detection of population size changes (Tajima's D, Fu & Li's F and D and Ramos-Onsins and Rozas R2) were calculated using DNAsp v6.10.04 [62]. The significance of all tests was determined by 10,000 coalescent simulations implemented in DNAsp. Highly significant negative values of Fs and small positive values of R2 indicate population growth whereas positive Fs values can result from balancing selection, population bottlenecks or the presence of population structure [63, 64]. Significantly negative values of Tajima’s D indicate recent range expansion (excess of low frequency polymorphisms), whereas significantly positive values are a signature of bottleneck or selection. A non-significant D is consistent with a population at drift-mutation equilibrium [64, 65].

## Results

### *Ceratomyxa* diversity as observed by light microscopy

**Fig 2:**
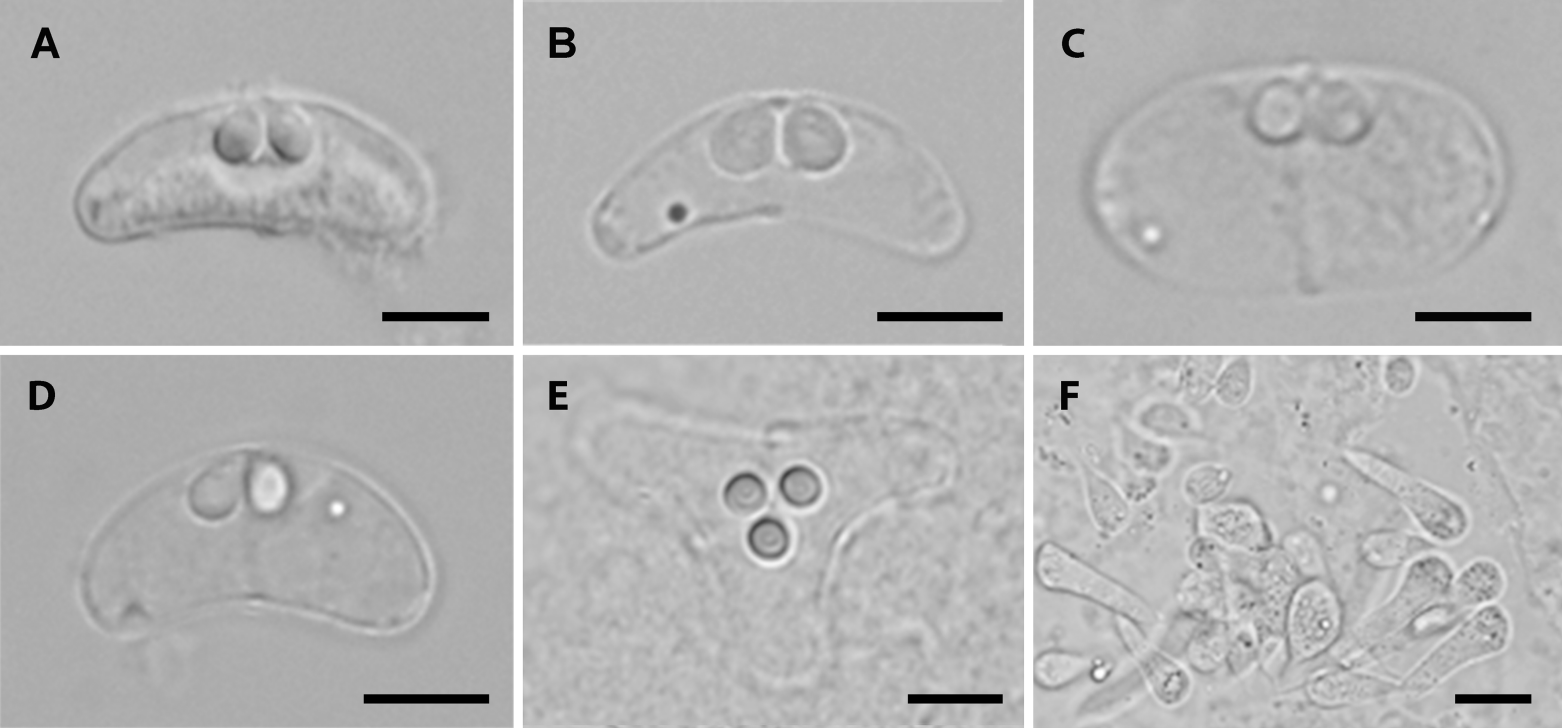
Light microscopy pictures of *Ceratomyxa* spp. spores of different morphotypes and plasmodia from clinid fish as seen in nomarski differential interference contrast. (A) *Ceratomyxa cottoidii* from *Clinus cottoides* from Herolds Bay. (B) *Ceratomyxa dehoopi* from *Clinus superciliosus* from Jacobs Bay. (C) *Ceratomyxa* sp. from *Clinus cottoides* from Jongensfontein. (D) *Ceratomyxa* sp. from *Muraenoclinus dorsalis* from Mouille Point. (E) Aberrant spore of *Ceratomyxa* sp. from *Clinus cottoides* from De Hoop. (F) Plasmodia of *Ceratomyxa* sp. from *Clinus cottoides* from Jongensfontein. Scale bar 5 μm (A–E), 20 μm (F).

Myxospores (Fig 2A–2E) and/or plasmodia (Fig 2F) of the genus *Ceratomyxa* were found in 40% of examined samples with the majority represented by infected gall bladders (58/152), and with few of them (3/152) found in bile ducts (liver). High morphological plasticity of *Ceratomyxa* spores was commonly observed in our samples with spore morphotypes similar to previously described *C*. *cottoidii* (Fig 2A) and *C*. *dehoopi* (Fig 2B) typical for their slender spores, however, oval round spores suggestive of novel species were also frequently seen (Fig 2C and 2D). Spore shape and size (length and thickness) varied even within single fresh samples. Aberrant *Ceratomyxa* spores with three polar capsules and three valves were occasionally observed (Fig 2E). Concurrent infections of *Ceratomyxa* with other myxozoan genera, *Sphaeromyxa* and *Kudoa* were also detected.

### Phylogenetic positioning of clinid ceratomyxids within the *Ceratomyxa* clade

**Fig 3:**
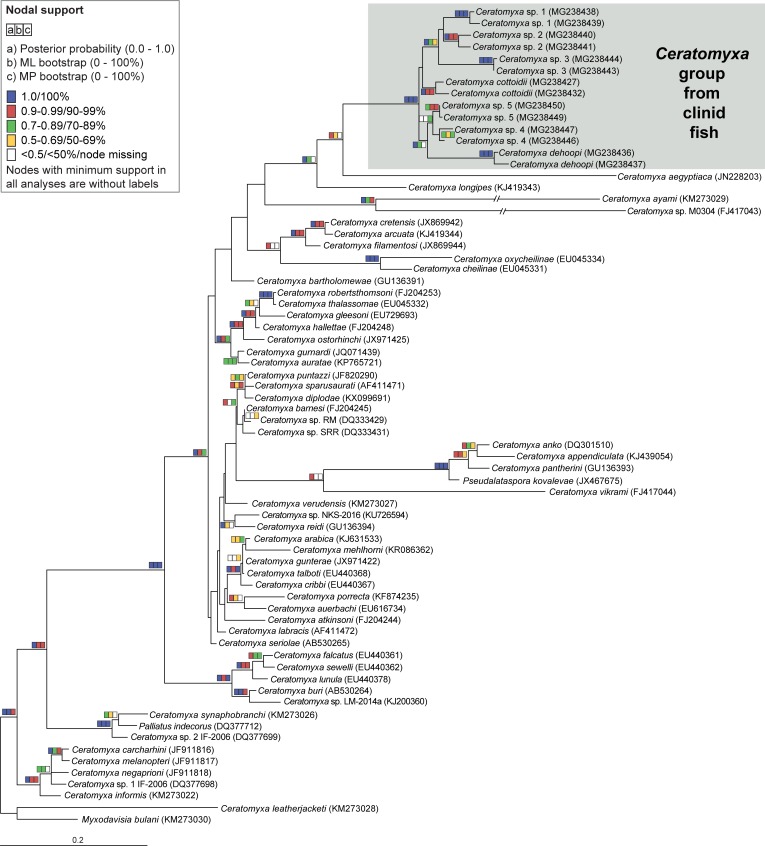
SSU rDNA-based maximum likelihood phylogenetic tree showing the position of clinid ceratomyxids within the *Ceratomyxa* clade. Nodal supports are shown for maximum likelihood and maximum parsimony bootstraps and Bayesian inference posterior probabilities. Branches of *Ceratomyxa ayami* and *Ceratomyxa* sp. M0304 were shortened to 50% of their original length. *Ceratomyxa leatherjacketi* and *Myxodavisia bulani* were used as outgroups.

The *Ceratomyxa* clade is an assemblage of diverse groups that evolved initially at different substitution rates and some of them have undergone subsequent radiation events [46]. This pattern is evident in ceratomyxids from South African clinids that clustered into a single well-supported long-branched lineage (“*Ceratomyxa* group from clinid fish“) which further split into several short-branched species (Fig 3). The whole lineage grouped with other extremely long-branching taxa with various scenarios in tree topology, most probably due to LBA artifacts. In ML and BI analyses (Fig 3), clinid ceratomyxids grouped with *Ceratomyxa aegyptiaca* from soleid fish from a Mediterranean coastal lagoon in northern Africa and *Ceratomyxa longipes* from a gadid fish sampled in the North Sea. The whole group then clustered either with a group including *Ceratomyxa ayami* and *Ceratomyxa* sp. M0304 (ML analysis, Fig 3) or formed a polytomy with two groups (*Ceratomyxa ayami* and *Ceratomyxa* sp. M0304; *C*. *anko*, *C*. *appendiculata*, *C*. *pantherini*, *C*. *vikrami* and *Pseudalataspora kovalevae*, BI analysis, BI posterior probability = 0.81). Another alternative in tree topology was observed in MP analysis, where clinid ceratomyxids grouped with *C*. *longipes* and then with the group formed by *C*. *anko*, *C*. *appendiculata*, *C*. *pantherini*, *C*. *vikrami* and *Pseudalataspora kovalevae* (MP bootstrap support <50%).

### Phylogenetic relationships within the clinid *Ceratomyxa* group

**Fig 4:**
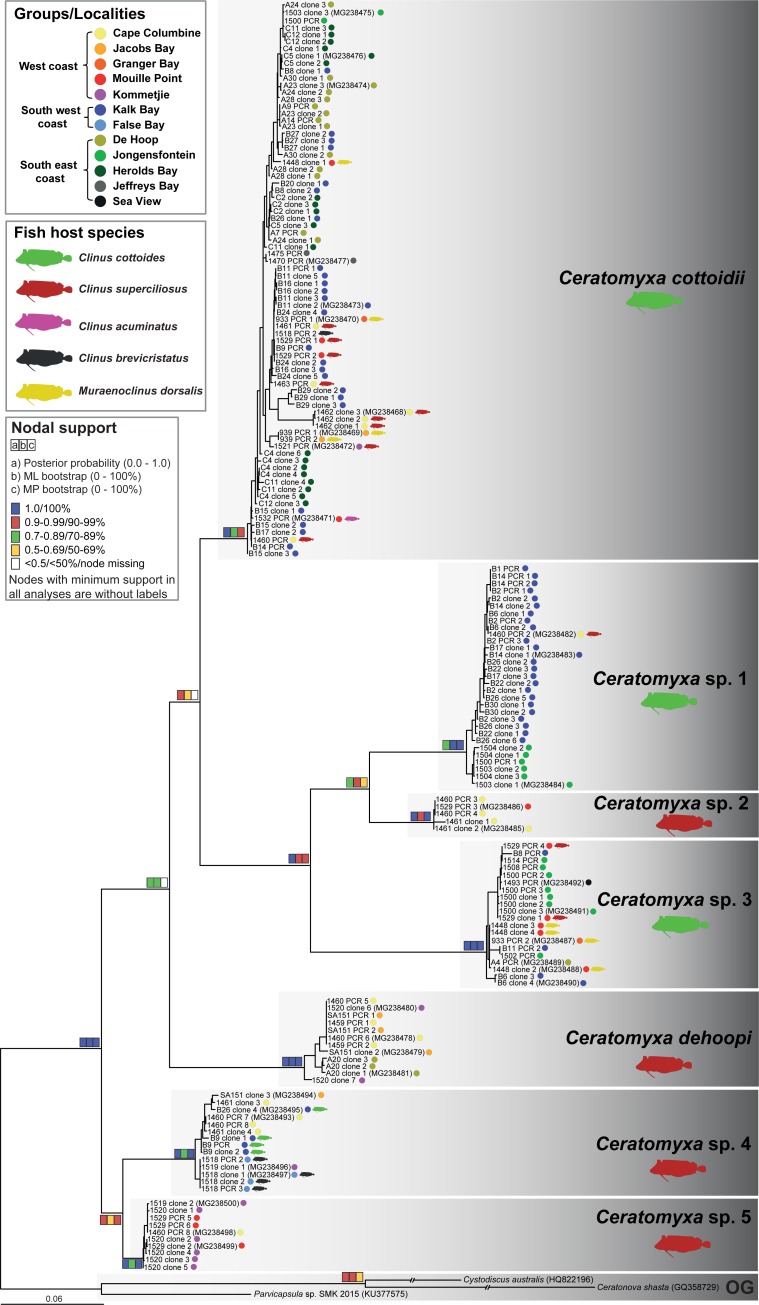
Maximum likelihood phylogenetic tree of the ITS region showing the relationships of *Ceratomyxa* spp. from South African clinids. Nodal supports are shown for maximum likelihood and maximum parsimony bootstraps and Bayesian inference posterior probability. *Cystodiscus australis*, *Ceratonova shasta* and *Parvicapsula* sp. were used as outgroups; branches of the first two outgroup taxa were shortened to 50% of their original length. Host species and localities are shown with colored symbols.

In this study, we amplified almost 400 clinid *Ceratomyxa* sequences belonging to different molecular markers of the nuclear ribosomal operon (SSU rDNA: n = 104; LSU rDNA: n = 118; Longer and shorter ITS region: n = 170, S1 Table) that were all used for subsequent phylogenetic analyses. After performing the analyses, unique sequence data representing each *Ceratomyxa* species from different fish hosts and localities were selected for GenBank submission (74 sequences, S2 Table). All other sequences are available in the S1 Dataset containing the untrimmed and trimmed fasta datasets of all molecular markers analyzed in this study. Blasting of newly obtained sequences against GenBank entries has shown that our data represent the first publicly available sequences of *Ceratomyxa* spp. from clinids. Phylogenetic analyses of SSU rDNA (S1 Fig) and ITS data (Fig 4) recognized seven well-supported novel lineages of *Ceratomyxa*, likely species, from South African clinids, whereas only five lineages were recovered in the LSU rDNA-based tree (S2 Fig) due to missing data from the two remaining lineages (*Ceratomyxa* sp. 2 and *Ceratomyxa* sp. 3). Based on the consensus of biological data (type host species and type locality) and, wherever possible, morphological data two of the lineages are considered to represent the previously described species *C*. *cottoidii* and *C*. *dehoopi*. The remainder of the sequences likely represents novel species (see paragraph “PCR screening, prevalence, distribution, co-infections and morphological remarks”). In the ITS-based tree, *C*. *cottoidii* clustered with a well-supported clade of *Ceratomyxa* sp. 1, *Ceratomyxa* sp. 2 and *Ceratomyxa* sp. 3 to create a common sister group to *C*. *dehoopi* (Fig 4). However, positioning of *C*. *cottoidii* and *C*. *dehoopi* varied depending on the marker used (S1 and S2 Figs). Another stable clade in all single gene analyses was formed by *Ceratomyxa* sp. 4 and *Ceratomyxa* sp. 5. Some members, i.e. *Ceratomyxa dehoopi* and *Ceratomyxa* sp. 3, created long branches to their sister taxa within the clinid *Ceratomyxa* group (Figs 3 and 4, S1 and S2 Figs).

### Species distances

**Fig 5:**
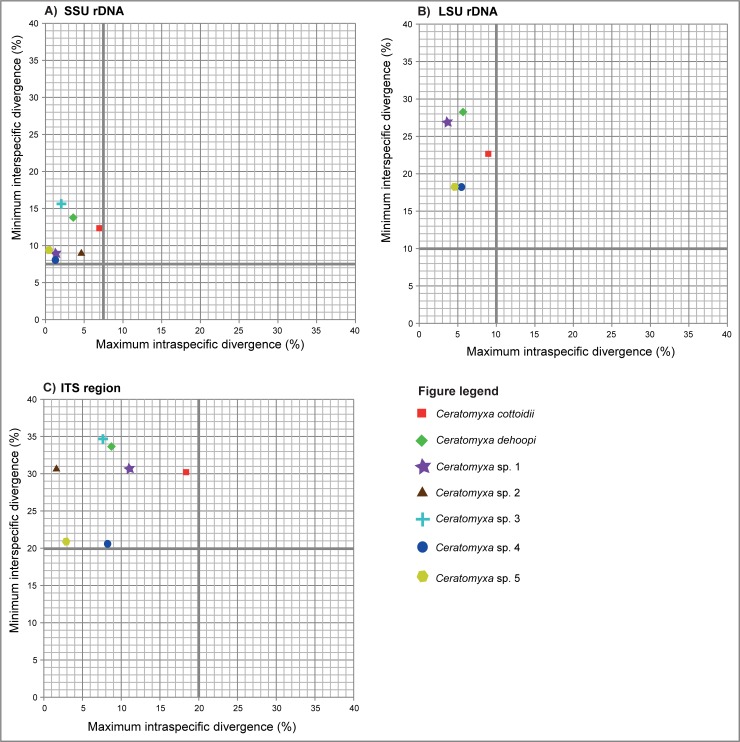
**Graphic interpretation of maximum intraspecific distances plotted against minimum interspecific distances (dissimilarities) among the sequences of (**A) SSU rDNA, (B) LSU rDNA and (C) ITS region of clinid *Ceratomyxa* spp. Intraspecific cut-off values (thresholds) are shown with thick grey-colored line.

SSU rDNA sequence variability among clinid ceratomyxids ranged from 7.8 to 15.7% whereas intraspecific divergence ranged from 0.4% in *Ceratomyxa* sp. 5 to the significantly higher level (7%) in *C*. *cottoidii* (Fig 5A). A similar trend was observed for the LSU rRNA gene with interspecific variation of 18.2–28.3% and intraspecific variation ranging from 4.7 to 9% (Fig 5B). The highest variation was calculated for the ITS region showing 20.9–33.6% interspecific divergence and 1.7–18.4% intraspecific distance (Fig 5C).

### PCR screening, prevalence, distribution, co-infections and morphological remarks

**Fig 6:**
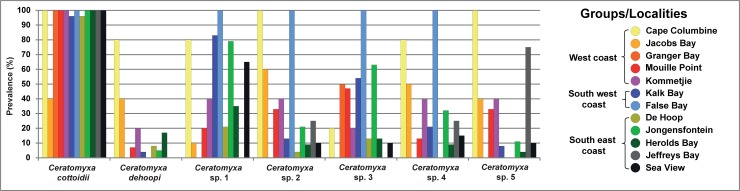
Prevalence of clinid *Ceratomyxa* spp. at each locality based on the PCR-screening of the shorter ITS region of all samples.

Species-specific PCR screening revealed 100% of samples positive at least for one *Ceratomyxa* species compared to only 40% parasite prevalence determined based on morphological observations (see paragraph “*Ceratomyxa* diversity as observed by light microscopy”). By species-specific PCR, *C*. *cottoidii* was recognized to be a true generalist with a high prevalence in all fish host species including its type host *C*. *cottoides*, while general clinid *Ceratomyxa* PCR revealed its presence in four out of five fish host species (Table 2). It is widely distributed from the west (Cape Columbine) to the east coast (Sea View) of South Africa with almost 100% prevalence at all studied localities. The only exception is its lower prevalence in Jacobs Bay (west coast) (Fig 6) where it was missing in all *C*. *superciliosus* and present in 80% of *M*. *dorsalis* samples. However, *C*. *superciliosus* was a common host of *C*. *cottoidii* at other localities (Table 2). On the other side, *C*. *dehoopi* is a parasite with a narrower host range (three out of five clinid species) and with the highest prevalence in *C*. *superciliosus* (Table 2). Its distributional range spans from Cape Columbine (west coast) to Herolds Bay (south coast). It was not detected at any of the two east coast localities (Fig 6). The remainder of *Ceratomyxa* spp. is represented by generalists, present in 4–5 examined fish species and distributed from the west to the east coast of South Africa with variable prevalence at each locality (Table 2, Fig 6). Importantly, the application of species-specific PCR was especially crucial for the assessment of the fish host species spectra of *C*. *dehoopi*, *Ceratomyxa* sp. 1 and *Ceratomyxa* sp. 5 for which general clinid *Ceratomyxa* PCR amplification was suggestive of their strict host specificity only in one fish species (Table 2).

**Table 2:** Prevalence of studied *Ceratomyxa* spp. in the fish host samples identified by PCR with general *Ceratomyxa* SSU rDNA, LSU rDNA and longer ITS region primers and species-specific shorter ITS-based PCR screening.

Myxozoan species	Marker	*Clinus cottoides*	*Clinus superciliosus*	*Clinus acuminatus*	*Clinus brevicristatus*	*Muraenoclinus dorsalis*
*Ceratomyxa cottoidii*	SSU rDNA	80% (31/39)	22% (2/9)	100% (4/4)	0% (0/1)	67% (2/3)
	LSU rDNA	86% (18/21)	14% (1/7)	100% (2/2)	0% (0/1)	100% (1/1)
	ITS region	71% (20/28)	14% (1/7)	N/A	0% (0/1)	100% (1/1)
	Screening	98% (110/112)	67% (12/18)	100% (6/6)	100% (1/1)	93% (14/15)
*Ceratomyxa dehoopi*	SSU rDNA	0% (0/39)	33% (3/9)	0% (0/4)	0% (0/1)	0% (0/3)
	LSU rDNA	0% (0/21)	29% (2/7)	0% (0/2)	0% (0/1)	0% (0/1)
	ITS region	0% (0/28)	43% (3/7)	N/A	0% (0/1)	0% (0/1)
	Screening	6% (7/112)	56% (10/18)	17% (1/6)	0% (0/1)	0% (0/15)
*Ceratomyxa* sp. 1	SSU rDNA	21% (8/39)	0% (0/9)	0% (0/4)	0% (0/1)	0% (0/3)
	LSU rDNA	38% (8/21)	0% (0/7)	0% (0/2)	0% (0/1)	0% (0/1)
	ITS region	32% (9/28)	0% (0/7)	N/A	0% (0/1)	0% (0/1)
	Screening	55% (61/112)	39% (7/18)	17% (1/6)	100% (1/1)	13% (2/15)
*Ceratomyxa* sp. 2	SSU rDNA	3% (1/39)	33% (3/9)	0% (0/4)	0% (0/1)	0% (0/3)
	LSU rDNA	0% (0/21)	0% (0/7)	0% (0/2)	0% (0/1)	0% (0/1)
	ITS region	0% (0/28)	14% (1/7)	N/A	0% (0/1)	0% (0/1)
	Screening	12% (13/112)	83% (15/18)	17% (1/6)	100% (1/1)	13% (2/15)
*Ceratomyxa* sp. 3	SSU rDNA	10% (4/39)	0% (0/9)	0% (0/4)	0% (0/1)	33% (1/3)
	LSU rDNA	0% (0/21)	0% (0/7)	0% (0/2)	0% (0/1)	0% (0/1)
	ITS region	7% (2/28)	14% (1/7)	N/A	0% (0/1)	100% (1/1)
	Screening	29% (32/112)	22% (4/18)	0% (0/6)	100% (1/1)	47% (7/15)
*Ceratomyxa* sp. 4	SSU rDNA	3% (1/39)	11% (1/9)	0% (0/4)	100% (1/1)	0% (0/3)
	LSU rDNA	10% (2/21)	29% (2/7)	0% (0/2)	100% (1/1)	0% (0/1)
	ITS region	7% (2/28)	43% (3/7)	N/A	100% (1/1)	0% (0/1)
	Screening	14% (16/112)	67% (12/18)	17% (1/6)	100% (1/1)	7% (1/15)
*Ceratomyxa* sp. 5	SSU rDNA	0% (0/39)	22% (2/9)	0% (0/4)	0% (0/1)	0% (0/3)
	LSU rDNA	0% (0/21)	43% (3/7)	0% (0/2)	0% (0/1)	0% (0/1)
	ITS region	0% (0/28)	43% (3/7)	N/A	0% (0/1)	0% (0/1)
	Screening	9% (10/112)	61% (11/18)	33% (2/6)	0% (0/1)	20% (3/15)

N/A: PCR not done.

Co-infections of single fish host samples by several *Ceratomyxa* species were extremely common and occurred in 71% of all samples. One sample from *C*. *superciliosus* from Cape Columbine even contained all *Ceratomyxa* spp. which was confirmed by variable shapes and dimensions of myxospores observed in this sample. On the other hand, many samples with single spore morphotype observed by LM showed multiple species infections. Single infections (29% of all samples) were mainly represented by *C*. *cottoidii* (42 samples) and only two samples exclusively contained *C*. *dehoopi* or *Ceratomyxa* sp. 5.

We were not able to draw any conclusions regarding the measurements of *C*. *cottoidii* spores from the original species description (Reed et al. 2007) and from our samples with single infections, for which LM measurements were taken, as the comparison would be biased by potential spore shrinkage of our ethanol-fixed material (details on spore measurements in S3 Table). Unfortunately, the lack of any samples with single infections of *C*. *dehoopi* and *Ceratomyxa* sp. 1–5 accompanied by spore measurements prevented analysis of spore morphometry for species comparison/descriptions. Therefore, *Ceratomyxa* sp. 1–5 currently represent phylogenetic lineages and remain undescribed species.

### Haplotype networks of *Ceratomyxa cottoidii* populations

**Fig 7:**
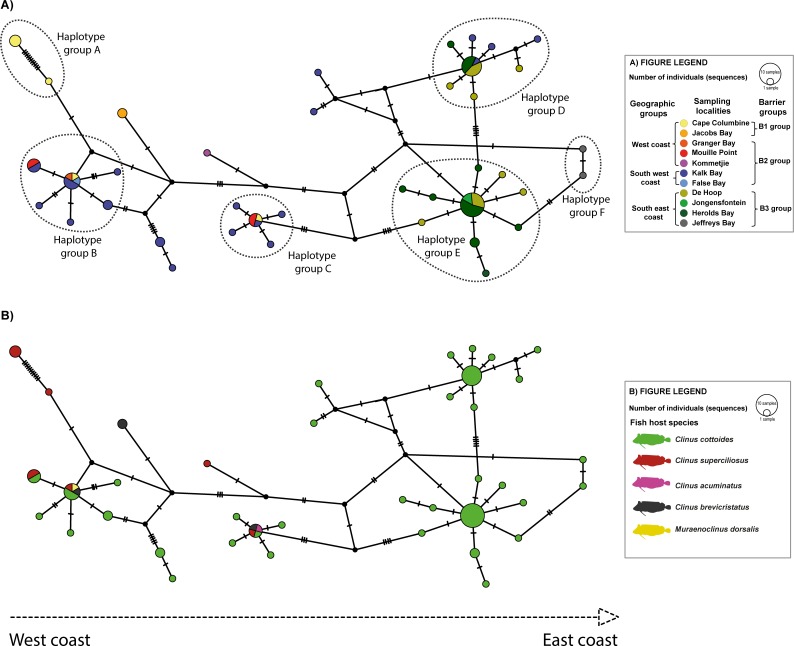
Haplotype network of *Ceratomyxa cottoidii* ITS dataset calculated using statistical parsimony networks (TCS) in PopArt v1.7. Haplotype network with (A) sampling localities and (B) host species used as traits. The sizes of haplotypic nodes are relative to the sample size. Each line represents one mutational step and dots represent alternative one-step mutations.

The haplotype network of *C*. *cottoidii* ITS region sequences with traits defined as geographic localities was represented by 39 haplotypes and showed a considerable complexity of haplotype grouping (Fig 7A). Isolates of *C*. *cottoidii* fell into six main haplotype groups. Haplotype group A, separated from the rest of the network by 11 mutations (nucleotide changes), was represented by samples from Cape Columbine, the most western locality of our sampling range. Haplotype groups B and C were mainly created by samples from Kalk Bay (south west coast) mixed with samples from the western localities. Haplotype group D was mainly represented by samples from De Hoop and Herolds Bay (south east coast) mixed with samples from the south-western locality Kalk Bay. Haplotype group E contained exclusively samples from the south-eastern coast. Two haplotypes from the south-eastern coast locality Jeffreys Bay formed haplotype group F separated from haplotype group E by two mutations. Haplotype groups B–E showed a radial branching pattern with the main haplotype surrounded by satellite low frequency haplotypes which is typical for young expanding populations. Due to small sample size (4 and 2 sequences, respectively), existence of a similar burst pattern cannot be excluded for haplotype groups A and F (Fig 7A).

The separation of haplotypes according to the fish host species was not evident in our analyses. Haplotypes from different fish species mixed with each other and the only two unmixed groups of parasite haplotypes were biased by their exclusive origin from a single host species (*C*. *cottoides*) sampled in Kalk Bay, De Hoop, Jongensfontein, Herolds Bay, and Jeffreys Bay, localities from which data from other host species were not available (Fig 7B).

### Population genetic structure of *Ceratomyxa cottoidii*

Approximately half (55.4%) of the variation in *C*. *cottoidii* was attributable to highly significant differences within populations whereas about 42.6% was associated with significant differences among geographic groups. On the other hand, very low variation was observed among populations (2.6%). The second AMOVA showed slightly higher variation among the barrier groups (50.0%) with highly significant covariance components. The within population variation was comparable with the one from the previous analysis. The lowest level of among group variation was found for AMOVA of host groups (26.0%), the analysis reaching highly significant levels only within and among populations (Table 3). In summary, our *a priori* AMOVA settings dividing *C*. *cottoidii* populations into groups delimited by major oceanographic barriers, especially if followed by the separation according to host gene flow barriers, explained significant amount of variation, however, additional factors except of fish host species, seem to impact population structuring of *C*. *cottoidii*. As revealed by AMOVA and haplotype networks, calculations of population genetics statistics for *C*. *cottoidii* using the dataset of parasite sequences from different fish species and their subsequent comparison with the population data from the type host *C*. *cottoides* is not a problem due to the lack of significant structuring of *C*. *cottoidii* populations according to fish host species.

**Table 3:** AMOVA partitioning of genetic variance for shorter ITS region within populations and groups of *Ceratomyxa cottoidii*. *A priori* groupings followed the separation of *C*. *cottoidii* sequences according to the i) geography of South African coastline (geographic groups), ii) main barriers to gene flow recognized for the populations of the fish host *Clinus cottoides* (barrier groups) and iii) fish host species (host groups).

% variation	Geographic groups	Barrier groups	Host groups
Among groups	42.58*	50.04**	26.02
Among populations	2.55	-3.06	25.35**
Within populations	55.36**	53.02**	48.63**

The covariance components are indicated as

** for highly significant (P < 0.01) and

* for significant (P < 0.05).

### Sequence diversity indices and demographic history of *Ceratomyxa cottoidii* populations

The differences between *C*. *cottoidii* populations based on geographic separation observed in haplotype networks and AMOVA were also evident in their population genetics statistics (Table 4). Results of DNA polymorphism statistical analysis showed generally high values of haplotype diversity with small differences between the analyzed groups. From the total of 39 haplotypes, the highest number of haplotypes was found in the south west (n = 20) and south east coast (n = 20) for the geography-related groups and in B2 (n = 22) for barrier groups. The nucleotide diversity was the lowest for the south east coast (resp. B3 group). Tajima's test showed significant negative values only for south east and B3 populations whereas Fu and Li's tests revealed significant negative values for the south west, south east coast, B2 and B3 populations. The R2 test was significant only for the south east coast and B3 group populations (Table 4). The results indicate that the south west, south east coast, B2 and B3 populations are expanding with the evidence of recent expansion in south east coast and B3 populations. In contrast, equilibrium and no population growth are suggested for the west and B1 populations at the edge of the distributional range of *C*. *cottoidii*.

**Table 4:** Results of population genetics statistics of ITS region for *Ceratomyxa cottoidii*.

Statistic tests	Geographic groups			Barrier groups		
	West coast	South west coast	South east coast	B1	B2	B3
Number of sequences (n)	14	25	37	8	31	37
Number of haplotypes (h)	7	20	20	5	22	20
Number of segregating sites (S)	27	38	20	23	38	20
Haplotype diversity (Hd)	0.901	0.983	0.908	0.857	0.972	0.908
Nucleotide diversity (π)	0.043	0.042	0.015	0.045	0.038	0.015
Tajima's D (D)	1.233	0.259	-0.688*	1.470	0.040	-0.688*
Fu and Li's D* (D*)	0.968	-1.030**	-1.512**	0.260	-1.743**	-1.512**
Fu and Li's F* (F*)	0.893	-0.785*	-1.453**	0.363	-1.408**	-1.453**
Ramos-Onsins & Rozas’ (R2)	0.197	0.125	0.086*	0.227	0.112	0.086*

The total number of unique haplotypes for all groups is 39; some haplotypes are shared by the groups. The statistical significance of covariance components is indicated as

** highly significant (P < 0.05) and

* significant (P < 0.1)

## Discussion

This is the first study to determine myxozoan diversity, distribution, prevalence and host specificity in a variety of clinid fish species, typical inhabitants of intertidal environments in South Africa. Moreover, the evolutionary history of the rapidly evolving clinid ceratomyxids has been assessed within this group as well as within the *Ceratomyxa* clade. Lastly, the most common clinid parasite in South Africa, *Ceratomyxa cottoidii*, has been subjected to population genetics analyses and its population structure has been compared with the data known for its type fish host, *Clinus cottoides*. This is a novel approach in myxozoan research as none of the previous studies compared the host-parasite genetic structure as they were solely focused on parasite population structuring [60, 66–69] and phylogeography [70–72].

We have revealed a high degree of species diversity represented by seven closely related *Ceratomyxa* spp. from the intertidal fishes Clinidae along the coast of South Africa. Such parasite radiation is probably the result of multiple speciation events triggered by limited host dispersal within this endemic area. Future application of a holistic approach in species descriptions based on detailed LM examination, proper photographic documentation and in-depth molecular identification is a good direction in accounting myxozoan species diversity, which currently seems to be underestimated [30, 73–76].

PCR screening with species-specific primers was crucial for the discovery of the remarkably high prevalence of ceratomyxids in clinid fish, with all examined samples infected with at least one parasite species and with some ceratomyxids even reaching 100% prevalence in certain hosts and at certain sites. Our findings underpin the rock pools as ideal habitats for promoting myxozoan life cycles and for the establishment of a diverse assemblage of organisms as reported previously for other animal groups [14–16]. Specific PCR assays were also essential for the exact assessment of *Ceratomyxa* spp. diversity in clinids, as frequent mixed infections, indeed common in myxozoans [17, 40], complicated such evaluation. Present evidence of multiple *Ceratomyxa* spp. infections indicates that the extreme spore variability observed in our samples, most likely resulted from concurrent infections of closely related species of different morphology. This may also be a case for *Ceratomyxa* sp. from *M*. *dorsalis* [37]. Moreover, high rates of mixed *Ceratomyxa* spp. infections encountered in such a restricted organ space are suggestive of a low or even lacking within-host competition in this niche. Nevertheless, a temporal and spatial separation of the infections during the host’s development may still occur similarly as reported for two competing marine myxozoan species [77].

In this study, we have unexpectedly revealed that *Ceratomyxa* spp. from South African clinids are not strictly host-specific parasites as a general rule for other ceratomyxids [25–30]. This may be caused by the close evolutionary relationships of South African clinids [8] and associated likely similar immune system traits of these fish as well as by generally low host immune response to coelozoic myxozoan parasites [78, 79]. We assume that several clinid fish species are true hosts for individual *Ceratomyxa* spp. as spores have been observed in the site of sporogony (gall bladder) at least in several samples in which the parasite has been molecularly detected.

The phylogenetic relationships of clinid *Ceratomyxa* spp. with other ceratomyxids were provided in this study. However, their accurate assessment was difficult due to their unstable clustering and weak nodal supports within the *Ceratomyxa* clade most probably due to LBA artifact. Nevertheless, increased taxon sampling with some closely related species, possibly from other blenniiform fishes, would facilitate to break the long-branch [46, 80] and thus improve inferring the exact positioning of this fast-evolving parasite group.

There is no universal criterion regarding what constitutes a sufficient level of sequence variation to represent distinct species in the genus *Ceratomyxa*. The intraspecific threshold values used for clinid ceratomyxids in present study are higher than commonly reported in other myxozoan clades [17] but they have been carefully evaluated with regard to the fast evolution of the studied molecular markers in this long-branched parasite group and we recommend using these cut-offs for this specific group of ceratomyxids in future studies. For example, relatively low values of maximum SSU rDNA intraspecific divergences were reported for ceratomyxids from Indian (up to 0.8%) [81], Australian (up to 1.3%) [26, 27, 30] and North Atlantic fishes (up to 1.6%) [46], whereas wide ranges up to 7% in *C*. *cottoidii* were encountered for South African clinid ceratomyxids (present study). Such large intraspecific variability of *C*. *cottoidii* may be a result of fast evolution of its genes concordant with increased substitution rates and associated tree branch lengths [82]. Therefore, settings of the intraspecific cut-offs and the following assessment of intraspecific and interspecific variation of a molecular marker have to be taken into account in relation to the substitution rate in a particular *Ceratomyxa* group. As evolutionary rates also vary for other myxozoan lineages, investigations into the sequence variation for a particular phylogenetic group are crucial for the assessment of the species concept in the Myxozoa. Moreover, it would be desirable to assess the intragenomic variation of ribosomal sequences for each myxozoan species from one individual (spore), however, low DNA yields associated with single spore extractions are limiting factors for such approach. Information about the intragenomic variation would be of interest also in the present population genetics analysis of *C*. *cottoidii*, however, distinguishing of multiple haplotype infections from the real intragenomic variability was not possible due to the fact that DNA was extracted from hundreds of spores from each infected fish individual. Moreover, spores in each host specimen may have been a genetic mixture that developed after an infection from a mixed pool of invertebrate alternate hosts harboring different *C*. *cottoidii* haplotypes. It is highly probable that intragenomic variation exists in *C*. *cottoidii* but this would only have a significant effect on “within individual level of variation” that was not assessed in our analyses. An alternative how to get around the intragenomic variability issue would be the use of mitochondrial (maternally inherited) genes, for example cytochrome c oxidase I or cytochrome b, but this approach was unfortunately not feasible in our case as such genes are difficult to amplify in myxosporeans in general and lead to several misinterpretations due to occurrence of pseudogenes (numts) [73].

*C*. *cottoidii* was shown to represent the most common parasite of clinids with a widespread distribution along the South African coastline. Investigation of a potential host-driven isolation of *C*. *cottoidii* populations, impelled by the newly discovered generalist life history of this parasite, showed no evidence for separation of parasite haplotypes by fish hosts. However, a certain bias may exist as the largest haplotype groups were characterized by a single host species. On the other hand, population structuring based on geography-related groupings (geographic and barrier groups) showed important overlapping patterns with fish host population structure [11, 12] consistent with the hypothesis that reduced host dispersal, as typical for *C*. *cottoides* [10], enhances parasite community differentiation. *C*. *cottoidii* population structure is formed by young expanding populations as well as by older populations in equilibrium. The older populations are represented by haplotypes from B1 group, which includes the most western localities, most probably at the edge of the distributional range of *C*. *cottoidii*. In these peripheral populations, no haplotype mixture is evident in the associated haplotype group A, likely representing the populations under recent speciation, which further supports the clustering of Cape Columbine sample (nr. 1462) as a separate lineage within (Fig 4) or at the base of the *Ceratomyxa* clade (S1 and S2 Figs). The young expanding populations of *C*. *cottoidii* are mainly represented by haplotypes from groups B2 and B3 covering western, south-western and south-eastern South African localities. Though some of the western localities are present in geographically mixed haplotype groups B and C (west + south west coast) and D (south west + south east coast), the west coast haplotypes never mixed with south east coast haplotypes. This pattern of population mixing does imply existence of some gene flow barriers on one side along with still ongoing migration between south west coast and south east coast locations on the other side, which is most probably facilitated by Agulhas ring eddies between Cape of Good Hope and Cape Agulhas in the zone of contact of Agulhas and Benguela current (Fig 1). Haplotype network suggests that some additional barrier to gene flow may exist within the south east coast populations (separation of haplotype group F), however, analysis of this hypothesis by splitting the south east group into south coast (De Hoop, Jongensfontein, Herolds Bay) and east coast (Jeffreys Bay) groups was not feasible due to low sample size from the eastern locality. Therefore, a more efficient fish taxon sampling, especially from the peripheral eastern and western localities, and following haplotype sequencing are necessary to support our conclusions which would additionally provide more data for detailed gene flow and migration analyses.

Our data suggest that oceanographic barriers around Cape Point, Cape Agulhas and east of Jeffreys Bay (east of Algoa Bay) region play important roles in the distribution and population structuring of the parasite as they do for the fish host *Clinus cottoides* [6, 11, 83]. This may be caused by closely linked host-parasite co-evolution in these space-limited habitats as genetic structure and co-divergence of host-parasite populations is higher in parasites infecting hosts with limited dispersal abilities [84]. However, only 43–50% of molecular variation in *C*. *cottoidii* sequences was ascribed to differences among groups associated with geography (Table 3) while a higher percentage of variation among groups (localities) was encountered for *C*. *cottoides* populations exhibiting significant oceanographic separation [11]. Such difference in variation may be linked to the complex myxozoan life cycle involving a vertebrate (intermediate) and an invertebrate (definitive) host, which adds another variable to the dispersal and associated population structure of *C*. *cottoidii*, a myxozoan with a presumably two-host life cycle [34]. *C*. *cottoides* is most commonly associated with mid-shore areas [2, 12], and while this fish host shows strong site fidelity, its ceratomyxid parasites may have a high dispersal capacity by distributing spores to other rock pools via tidal water exchange or wave splashes. An invertebrate host can be present in these rock pools or alternatively, it can inhabit other terrestrial or planktonic habitats and thus contributing further to the gene flow in *C*. *cottoidii* populations. The high infection prevalence in fish, however, suggests that the invertebrate host habitat overlaps with that of *C*. *cottoides*. Excellent candidates that are common in the intertidal rocky shore habitats are sedentary polychaetes (Annelida: Polychaeta) from the families Cirratulidae, Spionidae, Orbiniidae, Arenicolidae, Flabelligeridae, Sabellariidae, Terebellidae, Sabellidae, Serpulidae and Spirorbidae [52, 85]. A systematic approach specifically targeting the species present in rock pools in order to find myxozoan parasites within these is needed.

## Conclusions

We show that ceratomyxid species from South African clinids are a diverse group of fast evolving closely related parasites with high prevalence in their fish hosts, little host specificity and frequent concurrent infections, most probably as a result of radiation and no competition within the space limited host niche. *Ceratomyxa cottoidii* shows overlapping population structure with its type fish host, *C*. *cottoides*, however, data on the definitive host is required to unravel the complex network.

Several genetic studies of marine organisms, including *C*. *cottoides*, have shown that the sections of South African coastline coined as marine protected areas, representing hotspots of species richness and endemism and including a high diversity of habitats [86], require more protection [11, 83]. As both parasite diversity and distribution are closely linked to that of the host, any change in the conservation status and distribution of the host directly impacts that of the parasite and *vice versa* [87]. As *Ceratomyxa* parasites of clinid fishes are well represented in the South African marine fauna (present study), they may significantly impact their fish host populations. Investigations into the aquatic parasite biodiversity and distribution are of highest priority, as global climate change can shift the balance in healthy parasite-rich ecosystems where parasites represent one of the most susceptible groups to environmental change [87]. Besides the implications of global climate change for fish populations, a more detailed research of myxozoan life cycles as well as the roles of these parasites in food webs and trophic transfers and their impact on the health of fish hosts are desirable not only from a South African [23] but also from a global perspective.

## Supporting information

S1 Table List of clinid fish samples molecularly examined on the presence of *Ceratomyxa* spp. A detailed information on the host species and organ, locality, result of species-specific ITS-based PCR screening (+/-) and number of SSU rDNA, LSU rDNA and ITS PCR amplicons and/or clone sequences are available for each *Ceratomyxa* species along with the GenBank accession numbers (corresponding to those present in the phylogenetic trees). gb = gall bladder.
(XLSX)

S2 Table GenBank accession numbers of newly amplified *Ceratomyxa* sequences. (DOCX)

S3 Table List of spore measurements of *Ceratomyxa cottoidii* from the present study and from the original species description. (DOCX)

S1 Dataset The fasta datasets of untrimmed and trimmed molecular markers used for the phylogenetic analyses. (TXT)

S1 Fig Maximum likelihood phylogenetic tree of the SSU rDNA sequences showing the relationships of *Ceratomyxa* spp. from South African clinids. Nodal supports are mapped for maximum likelihood and maximum parsimony bootstraps and Bayesian inference posterior probabilities. *Ceratomyxa barnesi* and *Ceratomyxa longipes*, for which the branches were shortened to 50% of their original length, were used as outgroups. Host species and localities are shown with colored symbols.
(EPS)

S2 Fig Maximum likelihood phylogenetic tree of the LSU rDNA sequences showing the relationships of *Ceratomyxa* spp. from South African clinids. Nodal supports are mapped for maximum likelihood and maximum parsimony bootstraps and Bayesian inference posterior probabilities. *Ceratomyxa appendiculata*, *Ceratomyxa vikrami*, *Ceratomyxa cardinalis* and *Ceratomyxa verudaensis*, for which the branches were shortened to 50% of their original length, were used as outgroups. Host species and localities are shown with colored symbols.
(EPS)

## References

[1] Huggett J, Griffiths CL. Some relationships between elevation, physicochemical variables and biota of intertidal rock pools. Mar Ecol Prog Ser. 1986;29(2): 189–197.

[2] Bennett BA, Griffiths CL. Factors affecting the distribution, abundance and diversity of rock-pool fishes on the Cape Peninsula, South Africa. S Afr J Zool. 1984;19(2): 97–104.

[3] Prochazka K, Griffiths CL. The intertidal fish fauna of the west coast of South Africa—Species, community and biogeographic patterns. S Afr J Zool. 1992;27(3): 115–120.

[4] Teske PR, von der Heyden S, McQuaid CD, Barker NP. A review of marine phylogeography in southern Africa. S Afr J Sci. 2011;107(5–6): 43–53.

[5] von der Heyden S, Lipinski MR, Matthee CA. Remarkably low mtDNA control region diversity in an abundant demersal fish. Mol Phylogenet Evol. 2010;55(3): 1183–1188. doi: 10.1016/j.ympev.2009.09.018 19761857 10.1016/j.ympev.2009.09.018

[6] von der Heyden S, Bowie RCK, Prochazka K, Bloomer P, Crane NL, Bernardi G. Phylogeographic patterns and cryptic speciation across oceanographic barriers in South African intertidal fishes. J Evol Biol. 2011;24(11): 2505–2519. doi: 10.1111/j.1420-9101.2011.02382.x 21910777 10.1111/j.1420-9101.2011.02382.x

[7] Muller CM, von der Heyden S, Bowie RCK, Matthee CA. Oceanic circulation, local upwelling and palaeoclimatic changes linked to the phylogeography of the Cape sea urchin *Parechinus angulosus*. Mar Ecol Prog Ser. 2012;468: 203–215.

[8] Lin HC, Hastings PA. Phylogeny and biogeography of a shallow water fish clade (Teleostei: Blenniiformes). BMC Evol Biol. 2013;13: 18 doi: 10.1186/1471-2148-13-18 24067147 10.1186/1471-2148-13-210 PMC3849733

[9] Stepien CA. Evolution and biogeography of the Clinidae (Teleostei, Blennioidei). Copeia. 1992(2): 375–392.

[10] Wright D, Bishop JM, Matthee CA, von der Heyden S. Genetic isolation by distance reveals restricted dispersal across a range of life histories: Implications for biodiversity conservation planning across highly variable marine environments. Diversity and Distributions. 2015;21(6): 698–710.

[11] von der Heyden S, Prochazka K, Bowie RCK. Significant population structure and asymmetric gene flow patterns amidst expanding populations of *Clinus cottoides* (Perciformes, Clinidae): Application of molecular data to marine conservation planning in South Africa. Mol Ecol. 2008;17(22): 4812–4826. doi: 10.1111/j.1365-294X.2008.03959.x 19140974 10.1111/j.1365-294X.2008.03959.x

[12] von der Heyden S, Gildenhuys E, Bernardi G, Bowie R. Fine-scale biogeography: Tidal elevation strongly affects population genetic structure and demographic history in intertidal fishes. Front Biogeogr. 2013;5(1): 29–38.

[13] Holleman W, Von Der Heyden S, Zsilavecz G. Delineating the fishes of the *Clinus superciliosus* species complex in southern African waters (Blennioidei: Clinidae: Clinini), with the validation of *Clinus arborescens* Gilchrist & Thompson, 1908 and *Clinus ornatus* Gilchrist & Thompson, 1908, and with descriptions of two new species. Zool J Linnean Soc. 2012;166(4): 827–853.

[14] Firth LB, Schofield M, White FJ, Skov MW, Hawkins SJ. Biodiversity in intertidal rock pools: Informing engineering criteria for artificial habitat enhancement in the built environment. Mar Environ Res. 2014;102: 122–130. doi: 10.1016/j.marenvres.2014.03.016 24746927 10.1016/j.marenvres.2014.03.016

[15] Astles KL. Patterns of abundance and distribution of species in intertidal rock pools. J Mar Biol Assoc UK. 1993;73(3): 555–569.

[16] Mouritsen KN, Poulin R. Parasitism, community structure and biodiversity in intertidal ecosystems. Parasitology. 2002;124: S101–S117. 12396219 10.1017/s0031182002001476

[17] Okamura B, Gruhl A, Bartholomew JL. Myxozoan Evolution, Ecology and Development. Cham Heidelberg New York Dordrecht London: Springer; 2015.

[18] Fantham H. Some parasitic Protozoa found in South Africa II. S Afr J Sci. 1919;16: 185–191.

[19] Fantham H. Some parasitic Protozoa found in South Africa XIII. S Afr J Sci. 1930;27: 376–390.

[20] Reed CC, Basson L, Van As LL, Dyková I. Four new myxozoans (Myxosporea: Bivalvulida) from intertidal fishes along the south coast of Africa. Folia Parasitol. 2007;54(4): 283–292. 18303770 10.14411/fp.2007.037

[21] Bartošová-Sojková P, Kodádková A, Pecková H, Kuchta R, Reed CC. Morphology and phylogeny of two new species of S*phaeromyxa* Thélohan, 1892 (Cnidaria: Myxozoa) from marine fish (Clinidae and Trachichthyidae). Parasitology. 2015;142(5): 660–674. doi: 10.1017/S0031182014001723 25417699 10.1017/S0031182014001723

[22] Ali MA. *Ortholinea basma* n. sp (Myxozoa: Myxosporea) from Agile klipfish *Clinus agilis* (Teleostei: Clinidae), light and scanning electron microscopy. Eur J Protistol. 2000;36(1): 100–102.

[23] Smit NJ, Hadfield KA. Marine fish parasitology in South Africa: History of discovery and future direction. Af Zool. 2015;50(2): 79–92.

[24] Eiras J. Synopsis of the species of *Ceratomyxa* Thélohan, 1892 (Myxozoa: Myxosporea: Ceratomyxidae). Syst Parasitol. 2006:49–71. 10.1007/s11230-006-9039-5 16676229

[25] Gunter N, Adlard R. Bivalvulidan (Myxozoa: Myxosporea) parasites of damselfishes with description of twelve novel species from Australia's Great Barrier Reef. Parasitology. 2008;135(10): 1165–1178. doi: 10.1017/S0031182008004733 18771608 10.1017/S0031182008004733

[26] Gunter N, Whipps C, Adlard R. *Ceratomyxa* (Myxozoa: Bivalvulida): Robust taxon or genus of convenience? Int J Parasitol. 2009;39(12): 1395–1405. doi: 10.1016/j.ijpara.2009.04.008 19460381 10.1016/j.ijpara.2009.04.008

[27] Gunter N, Adlard R. Seven new species of *Ceratomyxa* Thélohan, 1892 (Myxozoa) from the gall-bladders of serranid fishes from the Great Barrier Reef, Australia. Syst Parasitol. 2009;73(1): 1–11. doi: 10.1007/s11230-008-9162-6 19337855 10.1007/s11230-008-9162-6

[28] Gunter N, Burger M, Adlard R. Morphometric and molecular characterisation of four new *Ceratomyxa* species (Myxosporea: Bivalvulida: Ceratomyxidae) from fishes off Lizard Island, Australia. Folia Parasitol. 2010: 1–10. 20449994 10.14411/fp.2010.001

[29] Alama-Bermejo G, Raga JA, Holzer AS. Host-parasite relationship of *Ceratomyxa puntazzi* n. sp (Myxozoa: Myxosporea) and sharpsnout seabream *Diplodus puntazzo* (Walbaum, 1792) from the Mediterranean with first data on ceratomyxid host specificity in sparids. Vet Parasitol. 2011;182(2–4): 181–192. doi: 10.1016/j.vetpar.2011.05.012 21680098 10.1016/j.vetpar.2011.05.012

[30] Heiniger H, Adlard RD. Molecular identification of cryptic species of *Ceratomyxa* Thélohan, 1892 (Myxosporea: Bivalvulida) including the description of eight novel species from apogonid fishes (Perciformes: Apogonidae) from Australian waters. Acta Parasitol. 2013;58(3): 342–360. doi: 10.2478/s11686-013-0149-3 23990433 10.2478/s11686-013-0149-3

[31] Sitjà-Bobadilla A, Alvarez-Pellitero P. Light and electron microscopic description of *Ceratomyxa labracis* n.sp. and redescription of *C*. *diplodae* (Myxosporea Bivalvulida) from wild and cultured Mediterranean sea bass (*Dicentrarchus labrax* L.). Syst Parasitol. 1993;26(3): 215–223.

[32] Alvarez-Pellitero P, Sitjà-Bobadilla A. Pathology of Myxosporea in marine fish culture. Dis Aquat Organ. 1993;17: 229–238.

[33] Palenzuela O, Sitjà-Bobadilla A, Alvarez-Pellitero P. *Ceratomyxa sparusaurati* (Protozoa: Myxosporea) infections in cultured gilthead sea bream *Sparus aurata* (Pisces: Teleostei) from Spain: Aspects of the host parasite relationship. Parasitol Res. 1997;83(6): 539–548. 9211504 10.1007/s004360050295

[34] Køie M, Karlsbakk E, Nylund A. The marine herring myxozoan *Ceratomyxa auerbachi* (Myxozoa: Ceratomyxidae) uses *Chone infundibuliformis* (Annelida: Polychaeta: Sabellidae) as invertebrate host. Folia Parasitol. 2008;55(2): 100–104. 18666412 10.14411/fp.2008.013

[35] Evans BS, Sweijd NA, Bowie RCK, Cook PA, Elliott NG. Population genetic structure of the perlemoen *Haliotis midae* in South Africa: Evidence of range expansion and founder events. Mar Ecol Prog Ser. 2004;270: 163–172.

[36] Teske PR, McQuaid CD, Froneman PW, Barker NP. Impacts of marine biogeographic boundaries on phylogeographic patterns of three South African estuarine crustaceans. Mar Ecol Prog Ser. 2006;314: 283–293.

[37] Tang L. Observations on myxozoans (Myzozoa: Myxosporea) and the spatial and temporal variation in parasite assemblages of the nosestripe klipfish, Muraenoclinus dorsalis Bleeke, 1860 (Perciformes: Clinidae). M.Sc. Thesis, University of Cape Town, Faculty of Science; 2010. Available from: https://open.uct.ac.za/bitstream/handle/11427/6210/thesis_sci_2010_tang_laura.pdf?sequence=1.

[38] Heiniger H, Gunter N, Adlard R. Relationships between four novel ceratomyxid parasites from the gall bladders of labrid fishes from Heron Island, Queensland, Australia. Parasitol Int. 2008;57(2): 158–165. doi: 10.1016/j.parint.2007.11.006 18252274 10.1016/j.parint.2007.11.006

[39] Morrison CM, Martell DJ, Leggiadro C, Oneil D. Ceratomyxa drepanopsettae in the gallbladder of Atlantic halibut, Hippoglossus hippoglossus, from the northwest Atlantic Ocean. Folia Parasitol. 1996;43(1): 20–36. 8682407

[40] Rocha S, Rangel LF, Castro R, Severino R, Azevedo C, Santos MJ, et al Ultrastructure and phylogeny of *Ceratomyxa diplodae* (Myxosporea: Ceratomyxidae), from gall bladder of European seabass *Dicentrarchus labrax*. Dis Aquat Organ. 2016;121(2): 117–128. doi: 10.3354/dao03049 27667809 10.3354/dao03049

[41] Evans NM, Holder MT, Barbeitos MS, Okamura B, Cartwright P. The phylogenetic position of Myxozoa: Exploring conflicting signals in phylogenomic and ribosomal data sets. Mol Biol Evol. 2010;27(12): 2733–2746. doi: 10.1093/molbev/msq159 20576761 10.1093/molbev/msq159

[42] Hartigan A, Estensoro I, Vancová M, Bílý T, Patra S, Eszterbauer E, et al New cell motility model observed in parasitic cnidarian *Sphaerospora molnari* (Myxozoa: Myxosporea) blood stages in fish. Sci Rep. 2016;6. 10.1038/srep39093 PMC5159882 27982057

[43] Castro LR, Austin AD, Dowton M. Contrasting rates of mitochondrial molecular evolution in parasitic diptera and hymenoptera. Mol Biol Evol. 2002;19(7): 1100–1113. doi: 10.1093/oxfordjournals.molbev.a004168 12082129 10.1093/oxfordjournals.molbev.a004168

[44] Eo SH, DeWoody JA. Evolutionary rates of mitochondrial genomes correspond to diversification rates and to contemporary species richness in birds and reptiles. Proc R Soc Lond B Biol Sci. 2010;277(1700): 3587–3592. 10.1098/rspb.2010.0965 PMC2982251 20610427

[45] Fiala I. The phylogeny of Myxosporea (Myxozoa) based on small subunit ribosomal RNA gene analysis. Int J Parasitol. 2006;36(14): 1521–1534. doi: 10.1016/j.ijpara.2006.06.016 16904677 10.1016/j.ijpara.2006.06.016

[46] Fiala I, Hlavničková M, Kodádková A, Freeman MA, Bartošová-Sojková P, Atkinson SD. Evolutionary origin of *Ceratonova shasta* and phylogeny of the marine myxosporean lineage. Mol Phylogenet Evol. 2015;86: 75–89. doi: 10.1016/j.ympev.2015.03.004 25797924 10.1016/j.ympev.2015.03.004

[47] Brandt M, Fischer-Blass B, Heinze J, Foitzik S. Population structure and the co-evolution between social parasites and their hosts. Mol Ecol. 2007;16(10): 2063–2078. doi: 10.1111/j.1365-294X.2007.03300.x 17498232 10.1111/j.1365-294X.2007.03300.x

[48] Pal C, Macia MD, Oliver A, Schachar I, Buckling A. Coevolution with viruses drives the evolution of bacterial mutation rates. Nature. 2007;450(7172): 1079–1081. doi: 10.1038/nature06350 18059461 10.1038/nature06350

[49] Papkou A, Gokhale CS, Traulsen A, Schulenburg H. Host-parasite coevolution: Why changing population size matters. Zoology. 2016;119(4): 330–338. doi: 10.1016/j.zool.2016.02.001 27161157 10.1016/j.zool.2016.02.001

[50] Patra S, Bartošová-Sojková P, Pecková H, Fiala I, Eszterbauer E. Holzer AS. Biodiversity and host-parasite cophylogeny of *Sphaerospora sensu stricto* (Cnidaria: Myxozoa). Under revision in Parasit Vectors. 10.1186/s13071-018-2863-z PMC6002976 29903034

[51] Holzer AS, Bartošová-Sojková P, Born-Torrijos A, Lövy A, Hartigan A, Fiala I. Joint evolution of myxozoa and their alternate hosts: a recipe for success and vast biodiversity? Under revision in Mol Ecol. 10.1111/mec.14558 29575260

[52] Branch GM, Griffiths CL, Branch ML, Beckley LE. Two Oceans: A Guide to the marine life of southern Africa. 1st ed. Claremont, South Africa: David Philip Publishers; 1994.

[53] Barta J, Martin D, Liberator P, Dashkevicz M, Anderson J, Feighner S, et al Phylogenetic relationships among eight *Eimeria* species infecting domestic fowl inferred using complete small subunit ribosomal DNA sequences. J Parasitol. 1997;83(2): 262–271. 9105308

[54] Katoh K, Misawa K, Kuma K, Miyata T. MAFFT: a novel method for rapid multiple sequence alignment based on fast Fourier transform. Nucleic Acids Res. 2002;30(14): 3059–3066. 12136088 10.1093/nar/gkf436 PMC135756

[55] Kearse M, Moir R, Wilson A, Stones-Havas S, Cheung M, Sturrock S, et al Geneious Basic: An integrated and extendable desktop software platform for the organization and analysis of sequence data. Bioinformatics. 2012;28(12): 1647–1649. doi: 10.1093/bioinformatics/bts199 22543367 10.1093/bioinformatics/bts199 PMC3371832

[56] Swofford DL. PAUP*. Phylogenetic Analysis Using Parsimony (*and Other Methods). Version 4. Sinauer Associates, Sunderland, Massachusetts 2003.

[57] Stamatakis A. RAxML-VI-HPC: Maximum likelihood-based phylogenetic analyses with thousands of taxa and mixed models. Bioinformatics. 2006;22(21): 2688–2690. doi: 10.1093/bioinformatics/btl446 16928733 10.1093/bioinformatics/btl446

[58] Ronquist F, Huelsenbeck JP. MrBayes 3: Bayesian phylogenetic inference under mixed models. Bioinformatics. 2003;19(12): 1572–1574. 12912839 10.1093/bioinformatics/btg180

[59] Rambaut A, Drummond AJ. Tracer v1.4; 2007. Available from: http://beast.bio.ed.ac.uk/Tracer.

[60] Lodh N, Kerans BL, Stevens L. The parasite that causes whirling disease, *Myxobolus cerebralis*, is genetically variable within and across spatial scales. J Euk Microbiol. 2012;59(1): 80–87. doi: 10.1111/j.1550-7408.2011.00596.x 22151695 10.1111/j.1550-7408.2011.00596.x

[61] Leigh JW, Bryant D. POPART: full-feature software for haplotype network construction. Method Ecol Evol. 2015;6(9): 1110–1116.

[62] Librado P, Rozas J. DnaSP v5: a software for comprehensive analysis of DNA polymorphism data. Bioinformatics. 2009;25(11): 1451–1452. doi: 10.1093/bioinformatics/btp187 19346325 10.1093/bioinformatics/btp187

[63] Fu YX. Statistical tests of neutrality of mutations against population growth, hitchhiking and background selection. Genetics. 1997;147(2): 915–925. 9335623 10.1093/genetics/147.2.915 PMC1208208

[64] Ramos-Onsins SE, Rozas J. Statistical properties of new neutrality tests against population growth. Mol Biol Evol. 2002;19(12): 2092–2100. doi: 10.1093/oxfordjournals.molbev.a004034 12446801 10.1093/oxfordjournals.molbev.a004034

[65] Tajima F. Statistical-method for testing the neutral mutation hypothesis by DNA polymorphism. Genetics. 1989;123(3): 585–595. 2513255 10.1093/genetics/123.3.585 PMC1203831

[66] Atkinson S, Bartholomew J. Disparate infection patterns of *Ceratomyxa shasta* (Myxozoa) in rainbow trout (*Oncorhynchus mykiss*) and Chinook salmon (*Oncorhynchus tshawytscha*) correlate with internal transcribed spacer-1 sequence variation in the parasite. Int J Parasitol. 2010: 599–604. doi: 10.1016/j.ijpara.2009.10.010 19895812 10.1016/j.ijpara.2009.10.010

[67] Atkinson SD, Bartholomew JL. Spatial, temporal and host factors structure the *Ceratomyxa shasta* (Myxozoa) population in the Klamath River basin. Infect Genet Evol. 2010;10(7): 1019–1026. doi: 10.1016/j.meegid.2010.06.013 20601174 10.1016/j.meegid.2010.06.013

[68] Atkinson SD, Jones SRM, Adlard RD, Bartholomew JL. Geographical and host distribution patterns of *Parvicapsula minibicornis* (Myxozoa) small subunit ribosomal RNA genetic types. Parasitology. 2011;138(8): 969–977. doi: 10.1017/S0031182011000734 21733258 10.1017/S0031182011000734

[69] Wuennemann H, Holzer AS, Pecková H, Bartošová-Sojková P, Eskens U, Lierz M. Repatriation of an old fish host as an opportunity for myxozoan parasite diversity: The example of the allis shad, *Alosa alosa* (Clupeidae), in the Rhine. Parasit Vectors. 2016;9. 10.1186/s13071-016-1760-6 PMC5024467 27628643

[70] Henderson M, Okamura B. The phylogeography of salmonid proliferative kidney disease in Europe and North America. Proc R Soc Lond B Biol Sci. 2004: 1729–1736. 10.1098/rspb.2004.2677 PMC1691782 15306294

[71] Whipps C, El-Matbouli M, Hedrick R, Blazer V, Kent M. *Myxobolus cerebralis* internal transcribed spacer 1 (ITS-1) sequences support recent spread of the parasite to North America and within Europe. Dis Aquat Organ. 2004: 105–108. doi: 10.3354/dao060105 15460854 10.3354/dao060105

[72] Whipps C, Kent M. Phylogeography of the cosmopolitan marine parasite *Kudoa thyrsites* (Myxozoa: Myxosporea). J Euk Microbiol. 2006: 364–373. doi: 10.1111/j.1550-7408.2006.00114.x 16968454 10.1111/j.1550-7408.2006.00114.x

[73] Bartošová P, Fiala I. Molecular evidence for the existence of cryptic species assemblages of several myxosporeans (Myxozoa). Parasitol Res. 2011;108(3): 573–583. doi: 10.1007/s00436-010-2100-y 20938686 10.1007/s00436-010-2100-y

[74] Bartošová P, Fiala I, Jirků M, Cinková M, Caffara M, Fioravanti ML, et al *Sphaerospora sensu stricto*: Taxonomy, diversity and evolution of a unique lineage of myxosporeans (Myxozoa). Mol Phylogenet Evol. 2013;68(1): 93–105. doi: 10.1016/j.ympev.2013.02.026 23500334 10.1016/j.ympev.2013.02.026

[75] Bartošová-Sojková P, Hrabcová M, Pecková H, Patra S, Kodádková A, Jurajda P, et al Hidden diversity and evolutionary trends in malacosporean parasites (Cnidaria: Myxozoa) identified using molecular phylogenetics. Int J Parasitol. 2014;44(8): 565–577. doi: 10.1016/j.ijpara.2014.04.005 24877770 10.1016/j.ijpara.2014.04.005

[76] Kodádková A, Dyková I, Tyml T, Ditrich O, Fiala I. Myxozoa in high Arctic: Survey on the central part of Svalbard archipelago. Int J Parasitol Parasites Wildl. 2014;3: 41–56. doi: 10.1016/j.ijppaw.2014.02.001 24918076 10.1016/j.ijppaw.2014.02.001 PMC4047956

[77] Holzer AS, Stewart S, Tildesley A, Wootten R, Sommerville C. Infection dynamics of two renal myxozoans in hatchery reared fry and juvenile Atlantic cod *Gadus morhua* L. Parasitology. 2010;137(10): 1501–1513. doi: 10.1017/S0031182010000247 20500915 10.1017/S0031182010000247

[78] Sitjà-Bobadilla A. Fish immune response to Myxozoan parasites. Parasite. 2008;15(3): 420–425. doi: 10.1051/parasite/2008153420 18814716 10.1051/parasite/2008153420

[79] Gomez D, Bartholomew J, Sunyer JO. Biology and mucosal immunity to myxozoans. Dev Comp Immunol. 2014;43(2): 243–256. doi: 10.1016/j.dci.2013.08.014 23994774 10.1016/j.dci.2013.08.014 PMC4216934

[80] Poe S. Evaluation of the strategy of long-branch subdivision to improve the accuracy of phylogenetic methods. Syst Biol. 2003;52(3): 423–428. 12775529 10.1080/10635150390197046

[81] Sanil NK, Chandran A, Shamal P, Binesh CP. Molecular and morphological descriptions of *Ceratomyxa collarae* n. sp and *Ceratomyxa leucosternoni* n. sp from marine ornamental fishes of Indian waters. Parasitol Res. 2017;116(2): 529–537. doi: 10.1007/s00436-016-5317-6 27885463 10.1007/s00436-016-5317-6

[82] Yang ZH. On the best evolutionary rate for phylogenetic analysis. Syst Biol. 1998;47(1): 125–133. 12064232 10.1080/106351598261067

[83] von der Heyden S. Why do we need to integrate population genetics into South African marine protected area planning? Afr J Mar Sci. 2009;31(2): 263–269.

[84] Blasco-Costa I, Poulin R. Host traits explain the genetic structure of parasites: a meta-analysis. Parasitology. 2013;140(10): 1316–1322. doi: 10.1017/S0031182013000784 23866918 10.1017/S0031182013000784

[85] Day JH. A monograph on the Polychaeta of Southern Africa. Part 2: Sedentaria. London: Trustees of the British Museum (Natural History); 1967.

[86] Blamey LK, Branch GM. Habitat diversity relative to wave action on rocky shores: Implications for the selection of marine protected areas. Aquatic Conserv Mar Freshw Ecosyst. 2008 Available from: http://www.academia.edu/11425285/Habitat_diversity_relative_to_wave_action_on_rocky_shores_implications_for_the_selection_of_marine_protected_areas.

[87] Adlard RD, Miller TL, Smit NJ. The butterfly effect: Parasite diversity, environment, and emerging disease in aquatic wildlife. Trends Parasitol. 2015;31(4): 160–166. doi: 10.1016/j.pt.2014.11.001 25488771 10.1016/j.pt.2014.11.001

