# Supplementary material for: Life in a rock pool: Radiation and population genetics of myxozoan parasites in hosts inhabiting restricted spaces
Source: PLoS One. 2018 Mar 21;13(3):e0194042. doi: 10.1371/journal.pone.0194042 (PMC5862482; doi:10.1371/journal.pone.0194042)
Supplement: S2 Table — (DOCX) [file pone.0194042.s002.docx]

**S2 Table. GenBank accession numbers of newly amplified *Ceratomyxa* sequences.**

| **Parasite species** | **Locality** | **Host species** | **SSU rDNA** | **LSU rDNA** | **ITS region** |
| --- | --- | --- | --- | --- | --- |
| *Ceratomyxa cottoidii* | Cape Columbine | *C. superciliosus* | MG238427 | MG238451 | MG238468 |
|  | Jacobs Bay | *M. dorsalis* | N/A | N/A | MG238469 |
|  | Granger Bay | *M. dorsalis* | N/A | N/A | MG238470 |
|  | Mouille Point | *C. acuminatus* | MG238428 | MG238452 | MG238471 |
|  | Kommetjie | *C. superciliosus* | N/A | N/A | MG238472 |
|  | Kalk Bay | *C. cottoides* | MG238429 | MG238453 | MG238473 |
|  | De Hoop | *C. cottoides* | MG238430 | MG238454 | MG238474 |
|  | Jongensfontein | *C. cottoides* | MG238431 | MG238455 | MG238475 |
|  | Herolds Bay | *C. cottoides* | MG238432 | N/A | MG238476 |
|  | Jeffreys Bay | *C. cottoides* | MG238433 | MG238456 | MG238477 |
|  | Sea View | *C. cottoides* | MG238434 | MG238457 | N/A |
| *Ceratomyxa dehoopi* | Cape Columbine | *C. superciliosus* | N/A | N/A | MG238478 |
|  | Jacobs Bay | *C. superciliosus* | MG238435 | MG238458 | MG238479 |
|  | Kommetjie | *C. superciliosus* | MG238436 | MG238459 | MG238480 |
|  | De Hoop | *C. superciliosus* | MG238437 | N/A | MG238481 |
| *Ceratomyxa* sp. 1 | Cape Columbine | *C. superciliosus* | N/A | N/A | MG238482 |
|  | Kalk Bay | *C. cottoides* | MG238438 | MG238460 | MG238483 |
|  | Jongensfontein | *C. cottoides* | MG238439 | MG238461 | MG238484 |
| *Ceratomyxa* sp. 2 | Cape Columbine | *C. superciliosus* | MG238440 | N/A | MG238485 |
|  | Mouille Point | *C. superciliosus* | MG238441 | N/A | MG238486 |
|  | Jongensfontein | *C. cottoides* | MG238442 | N/A | N/A |
| *Ceratomyxa* sp. 3 | Granger Bay | *M. dorsalis* | N/A | N/A | MG238487 |
|  | Mouille Point | *M. dorsalis* | MG238443 | N/A | MG238488 |
|  | De Hoop | *C. cottoides* | N/A | N/A | MG238489 |
|  | Kalk Bay | *C. cottoides* | MG238444 | N/A | MG238490 |
|  | Jongensfontein | *C. cottoides* | MG238445 | N/A | MG238491 |
|  | Sea View | *C. cottoides* | N/A | N/A | MG238492 |
| *Ceratomyxa* sp. 4 | Cape Columbine | *C. superciliosus* | MG238446 | MG238462 | MG238493 |
|  | Jacobs Bay | *C. superciliosus* | N/A | N/A | MG238494 |
|  | Kalk Bay | *C. cottoides* | N/A | MG238463 | MG238495 |
|  | Kommetjie | *C. superciliosus* | N/A | N/A | MG238496 |
|  | False Bay | *C. brevicristatus* | MG238447 | MG238464 | MG238497 |
|  | Jongensfontein | *C. cottoides* | MG238448 | MG238465 | N/A |
| *Ceratomyxa* sp. 5 | Cape Columbine | *C. superciliosus* | N/A | N/A | MG238498 |
|  | Mouille Point | *C. superciliosus* | MG238449 | MG238466 | MG238499 |
|  | Kommetjie | *C. superciliosus* | MG238450 | MG238467 | MG238500 |

N/A – sequence not available; *C. cottoides – Clinus cottoides*; *C. superciliosus* – *Clinus superciliosus*; *C. acuminatus* – *Clinus acuminatus*; *C. brevicristatus – Clinus brevicristatus*; *M. dorsalis – Muraenoclinus dorsalis.*
