# Supplementary material for: Life in a rock pool: Radiation and population genetics of myxozoan parasites in hosts inhabiting restricted spaces
Source: PLoS One. 2018 Mar 21;13(3):e0194042. doi: 10.1371/journal.pone.0194042 (PMC5862482; doi:10.1371/journal.pone.0194042)
Supplement: S3 Table — (DOCX) [file pone.0194042.s003.docx]

**S3** **Table. List of spore measurements of *Ceratomyxa cottoidii* from the present study and from the original species description.**

|  | Present study | Species description |
| --- | --- | --- |
| Spore length | 8.0 ± 0.8 (6.6–9.8) | 7.1 ± 0.6 (6.5–8.0) |
| Spore thickness | 17.0 ± 1.5 (14.9–21.1) | 18.2 ± 1.7 (17.0–22.0) |
| Polar capsule length | 2.6 ± 0.2 (2.1–2.9) | 2.7 ± 0.4 (2.3–3.0) |
| Polar capsule width | 2.0 ± 0.2 (1.7–2.4) | 2.4 ± 0.4 (2.0–3.0) |
| Posterior spore angle | 168º | Unknown |

Note: Arithmetic mean and standard deviation are provided followed by minimum and maximum values of spore measurements in parentheses (in micrometers). Species description (Reed et al. 2007) measurements were performed from live spores obtained from *Clinus cottoides* from De Hoop. In present study, the ethanol-fixed spores of *Ceratomyxa cottoidii* were measured from samples with single parasite infections (n = 13 samples, 46 spores) from the type host and type locality according to the guidelines of Lom and Arthur (1989) using ImageJ v.1_44p software (Wayne Rasband, http://imagej.nih.gov/ij/).

References

Lom J, Arthur J. A guideline for the preparation of species descriptions in Myxosporea. J Fish Dis. 1989;12(2): 151-156.
